# The complete genome sequence of five pre-2013 Escherichia coli sequence type (ST)1193 strains reveals insights into an emerging pathogen

**DOI:** 10.1099/acmi.0.000894.v3

**Published:** 2024-10-18

**Keywords:** antibiotic resistance, capsule, long-read sequencing, mobile genetic elements, phylogenetic analysis

## Abstract

Fluoroquinolone-resistant *Escherichia coli* sequence type (ST)1193 is a profound, emerging lineage associated with systemic, urinary tract and neonatal infections. Humans, companion animals and the environment are reservoirs for ST1193, which has been disseminated globally. Following its detection in 2007, ST1193 has been identified repeatedly amongst fluoroquinolone-resistant clones in Australia. However, despite the growing importance of ST1193, only three complete genomes are published in the literature, none of which are from Australia. Here we expand on the available ST1193 resources with the complete genomes of five ST1193 strains sequenced using Oxford Nanopore Technologies and Illumina. Using *in silico* genotyping, we found that all strains were multi-drug resistant, including resistances to fluoroquinolones and cephalosporins. *In vitro* antibiotic susceptibility testing mostly correlated with individual genotypes. The exception was MS8320, which had additional *in vitro* resistance to piperacillin/tazobactam, ampicillin/sulbactam, cefazolin and doripenem (carbapenem). Further investigation identified seven additional copies of an IS26 transposable unit carrying a *bla*_TEM-1B_ beta-lactamase gene, suggesting this tandem amplification is associated with extended resistance phenotypes. Uropathogenicity factors, including three separate siderophore-encoding loci, were conserved in chromosomal and plasmid regions. Using all complete genomes, we further elucidated the recombination events surrounding the previously described K5/K1 capsular locus switch. Phenotypic confirmation of differing capsules in Australian ST1193 strains, coupled with genetic analysis revealing insertions downstream of the capsular locus, underscored the genetic distinctions between K5 and K1 capsule encoding strains. This study provides five new reference ST1193 genomes from Australia. These include the earliest complete K5-capsule ST1193 genomes on record (collected 2007), alongside our reference genome (MS10858), a clinical isolate obtained early during the ST1193 expansion and representative of the predominant K1-associated clade. These findings lay the foundations for further genomic and molecular analyses that may help understand the underlying reasons for the rapid global expansion of ST1193.

**Impact Statement**

This study enhances the understanding of fluoroquinolone-resistant *Escherichia coli* sequence type (ST)1193, a growing concern in urinary tract infection management. By understanding the genomic background of fluoroquinolone resistance and predicting potential recombination events contributing to antibiotic resistance acquisition, we deliver insights into the evolutionary trajectory and dissemination of antibiotic resistance in ST1193. Through genomic analyses, including the generation of complete genomes for five representative strains, we broaden the ST1193 genomic dataset and reveal key virulence and antibiotic resistance gene content. Our findings can help guide therapeutic strategies, surveillance initiatives and infection control measures, aiming to alleviate the global health and economic ramifications of antibiotic-resistant *E. coli* infections.

## Data Summary

The study sequences are available in the National Center for Biotechnology Information (NCBI) under BioProject accession number PRJNA493523. The raw sequence read data generated in this study have been deposited in the NCBI sequence read archive (SRA; https://www.ncbi.nlm.nih.gov/sra) under accession numbers SRR7912362, SRR7912381, SRR7912383, SRR7912392, SRR7912411, and SRR12008669 to SRR12008673. The complete assemblies have been deposited in GenBank under accession numbers CP058897 to CP058900, CP058917 to CP058924, and CP059332 to CP059342. The software used to analyse raw sequence reads for polymorphism discovery and whole-genome sequencing-based phylogenetic reconstruction are available as described in the Methods. The authors confirm that all supporting data protocols have been provided in the article or supplementary data files.

## Introduction

Uropathogenic *Escherichia coli* (UPEC) are significant community- and hospital-acquired pathogens [1,2]. The emergence of multidrug resistance complicates treatment options and exacerbates the health and economic impacts of UPEC infections. Among the widely prescribed broad-spectrum antibiotics for urinary tract infections (UTIs), fluoroquinolones took precedence, accounting for 70% of UTI treatments by 2001 in some jurisdictions [3,4]. In Australia, despite restrictions on quinolone use in humans and regulations prohibiting their use in food-producing animals [5], there has been a notable increase in *E. coli* resistance to fluoroquinolones, particularly in the Northern Territory and Western Australia [6,7]. Globally, the extensive use of fluoroquinolones has driven the emergence of fluoroquinolone-resistant UPEC lineages across all World Health Organization (WHO) regions [8,9].

The development of fluoroquinolone resistance in UPEC can be rapid [10,15] and may result in prolonged or recurrent infection [16]. The globally disseminated *E. coli* sequence type (ST)131 is the most well-studied UPEC clonal lineage. High rates of resistance to fluoroquinolones (and co-resistance to extended-spectrum beta*-*lactam antibiotics) among ST131 probably facilitated its global dissemination over the past two decades [11,15, 17, 18].

Another emerging UPEC lineage, *E. coli* ST1193, has been associated with UTI [19], bloodstream infections [20] and neonatal invasive diseases [21,22]. Like *E. coli* ST131, ST1193 frequently exhibits multidrug resistance [23] and high rates of fluoroquinolone resistance. Platell *et al*. [19] were among the first to report clinical fluoroquinolone-resistant ST1193 among isolates collected from cases of UTI. Between 2007 and 2009, ST1193 represented 53% of non-ST131 fluoroquinolone-resistant isolates from humans and domesticated canines in Australia [19]. Since then, fluoroquinolone-resistant ST1193 have emerged and disseminated globally [13]. ST1193 has been isolated from humans [13,20, 22,31], companion animals [29,30, 32, 33] and the environment [34,35]. Unlike ST131, evidence for stepwise acquisition of single-nucleotide polymorphisms (SNPs) associated with fluoroquinolone resistance has not been established for ST1193. Instead, a contemporary study suggested that ST1193 acquired fluoroquinolone resistance as recently as 2005 through multiple recombination events with other antimicrobial-resistant *E. coli* clones [36].

Molecular genotyping of the O-antigen polysaccharide shows ST1193 is an O75-associated lineage belonging to clonal complex (CC)14 [23] within the *E. coli* phylogroup B2 [29,33]. A deletion event triggering a frameshift mutation in *lacY* (encoding lactose permease) has induced a lactose non-fermentation phenotype in ST1193 [33] and other CC14 strains [13]. This particular phenotype might have contributed to the under-reporting of ST1193 before 2010, possibly due to selective and differential use of clinical diagnostic tests relying on MacConkey agar [37].

The genetic landscape of ST1193 comprises two major clades: ‘K5-associated’ or ‘K1-associated’ ST1193 [13], which is related to differences in the capsular antigen. This capsule-switching phenomenon, from a K5 to a K1 antigen, probably underpins the rapid global expansion of ST1193 [13]. However, at the time of writing, only three complete ST1193 genomes (all ‘K1-associated’) were published in the literature, with two strains from humans [21,38], and one from river water [39]. To expand the genomic dataset and unravel the genomic content of ST1193, the genomes of three human-derived ST1193 strains with a K1 capsule and two strains with a K5 capsule underwent whole-genome sequencing (WGS) using both long-read and short-read sequencing. We generated complete genomes for all five ST1193 strains, including manual curation of mobile genetic elements, and defined the full complement and genomic context of virulence and antibiotic resistance genes. The addition of five complete ST1193 genomes across both capsule types will facilitate future comparative genomics studies of this important UPEC clone.

## Methods

### Bacterial strains

The five *E. coli* ST1193 strains analysed in this study were obtained from three different sources. One isolate (MS10711, Alternative ID: GNB 2889) originated from the Australian Group on Antimicrobial Resistance (AGAR), a collaboration between clinicians and microbiologists (www.agargroup.org, accessed 4 April 2024). The remaining four isolates were sourced from previously published studies [19,28]. Detailed information on the five ST1193 strains sequenced in this study are listed in Table S1 (available in the online version of this article).

### Phenotypic assays

To confirm the capsule type phenotypically, the cross-brush method was performed as previously described [40] using the K1 and K5 lytic phage sourced from the Statens Serum Institut (Denmark). After crossing the phage suspension line, growth inhibition was considered a positive reaction. For antibiotic susceptibility testing (AST), isolates were sent to The University of Queensland Centre for Clinical Research (UQCCR) for AST using a broth microdilution method (BMD) with *E. coli* strain ATCC 25922 as quality control. Cultures were inoculated from −80 °C storage onto Columbia Horse Blood Agar and incubated at 37 °C overnight. A 0.5 McFarland suspension was prepared in sterile water (ISO20776.1-2017). Eleven microlitres of suspension was added to 11 ml cation-adjusted Mueller Hinton Broth with TES buffer, and 50 µl of broth suspension was added to each well of a GN6F Sensititre Plate (Thermo Fisher). Plates were sealed and incubated at 35 °C for 19 h before determining the minimum inhibitory concentration (MIC) using the Thermo Scientific Sensititre Vizion system instrument with SWIN software v3.4. MICs and sensitive, intermediate and resistant (SIR) interpretations were reported according to European Committee on Antimicrobial Susceptibility Testing (EUCAST) and Clinical and Laboratory Standards Institute (CLSI) breakpoints (User Manual SWIN Software System, v3.4).

### Genomic DNA extraction, library preparation and sequencing

Bacteria were grown in lysogeny broth (LB), and genomic DNA was extracted using the MoBio UltraClean Microbial DNA isolation kit (as per the manufacturer’s instructions). Genome sequencing was performed using Illumina short-read and nanopore long-read sequencing as previously described [41,43]. We performed Illumina sequencing using the NextSeq 500 platform at the Australian Centre for Ecogenomics (University of Queensland, Brisbane, Australia). DNA libraries were prepared with the Nextera XT Library Preparation Kit and indexed with the Nextera XT Index Kit (both from Illumina). Sequencing generated 150 bp paired-end reads. The Oxford Nanopore Technologies (ONT) sequencing libraries were prepared using 400–600 ng of genomic DNA, according to the rapid barcoding sequencing kit (catalogue number SQK-RBK004, 12 barcodes mixed in equal volume) as per the manufacturer’s instructions. The entire library was loaded onto an R9.4 flow cell and run on a MinION device for approximately 48 h (using MinKNOW v1.10.16). The reads generated from the MinION sequencing run were basecalled and binned using the ONT Guppy basecalling suite v3.1.5 – Guppy basecaller and Guppy barcoder, respectively. Further details of methods including nanopore read quality control, Illumina read quality control and genome assembly are available in the Supplementary Materials.

### Genome annotations

The assemblies were annotated using Prokka v1.14.0 [44], with the complete genome of *E. coli* ST131 strain EC958 (GenBank: HG941718) as a reference for trusted proteins. Prophage regions were identified using PHASTER [45,46]. Mobile genetic elements were identified using IslandViewer 4 [47] and ISsaga v2.0 [48] (ISfinder platform [49]), followed by manual curation using Artemis v18.1.0 [50]. Methods for *in silico* multilocus sequence typing, genotyping the O, H and K-antigens (lipopolysaccharide, flagellar and capsule, respectively), along with virulence and antibiotic resistance gene genotyping (including chromosomal point mutations) have been described previously [15].

### Investigating regions of recombination

The complete chromosome sequences from the five ST1193 genomes sequenced in this study underwent a reference-free whole-chromosome alignment with three publicly available, complete ST1193 chromosomes using progressiveMauve [51] in Mauve (version snapshot_2015-02-13) [52]. SNPs were extracted from the whole-chromosome alignment using HarvestTools v1.2 [53]. To identify regions of recombination, the whole-chromosome alignment was input into Gubbins v2.4.1 [54] [default settings, ‘raxml mode’ with the General Time Reversible (GTR) GAMMA correction]. The predicted regions of recombination were visualized using the ggplot2 v3.3.5 library [55] in R v4.1.0 [56].

### Compiling a high-quality ST1193 global dataset and identifying genetic variants

We sequenced five Australian ST1193 genomes to compare them with other ST1193 genomes sampled worldwide. To identify additional ST1193 genomes, we first screened the Enterobase database v1.1.2 (https://enterobase.warwick.ac.uk/, accessed 21 October 2021) for strains belonging to ST1193. This screening identified 616 ST1193 genomes. We then downloaded the corresponding epidemiological data (e.g. collection date, source and country of origin) from Enterobase. Subsequently, we retrieved the sequence read data for these 616 ST1193 genomes from the National Center for Biotechnology Information (NCBI) sequence read archive (SRA) using the ‘prefetch’ and ‘fastq-dump’ tools within the SRA Toolkit v2.9.0-mac64 (http://ncbi.github.io/sra-toolshttps://github.com/ncbi/sra-tools, accessed 21 October 2021).

We integrated multiple complete chromosomes by simulating error-free reads using ART (version ART-MountRainier-2016-06-05) [57] to 60× coverage with an insert size of 340±40 bp. These included the three publicly available and published chromosomes [09-02E (GenBank: AP022650), AVS0096 (GenBank: CP076344) and MCJCHV-1 (GenBank: CP030111)]. A read-mapping approach for identifying genetic variants was performed using the SPANDx v3.2 pipeline, as previously described [58]. In brief, trimmed Illumina reads were mapped to the complete chromosome MS10858, originally isolated from a female with a urine infection in Australia in October 2007 [19]. Our final dataset consisted of 619 genomes representing previously published datasets (including three complete genomes) plus our Australian collection (*n*=5).

### High-resolution phylogeny of ST1193

The quality-trimmed paired-end Illumina reads from the 623 ST1193 genomes were mapped onto the chromosome of MS10858 as described above. SNPs within regions of high-density clusters (≥3 SNPs found within a 10 bp window) and predicted recombination sites (identified using Gubbins) were removed from the core-genome alignment. Core-genome SNP alignments were independently run through jModelTest v2.1.10 [59,60], to identify the best-fit evolutionary model using 12 candidate models from three substitution schemes with base tree for likelihood calculations optimized for maximum likelihood. jModelTest included models with equal/unequal base frequencies (+F), with/without a proportion of invariable sites (+I) and with/without rate variation among sites (+G) (four rate categories). Maximum likelihood phylogenetic trees were generated from the orthologous core-genome SNP alignments using RAxML v8.2.10 [61] (GTR correction) through optimization of ten distinct, randomized maximum parsimony trees, before adding 1000 bootstrap replicates. The resulting phylogenetic trees were visualized using FigTree v1.4.4 (http://tree.bio.ed.ac.uk/software/figtree/, accessed 8 April 2024).

## Results

### Genomic descriptions of complete ST1193 representative genomes

**Fig. 1.**
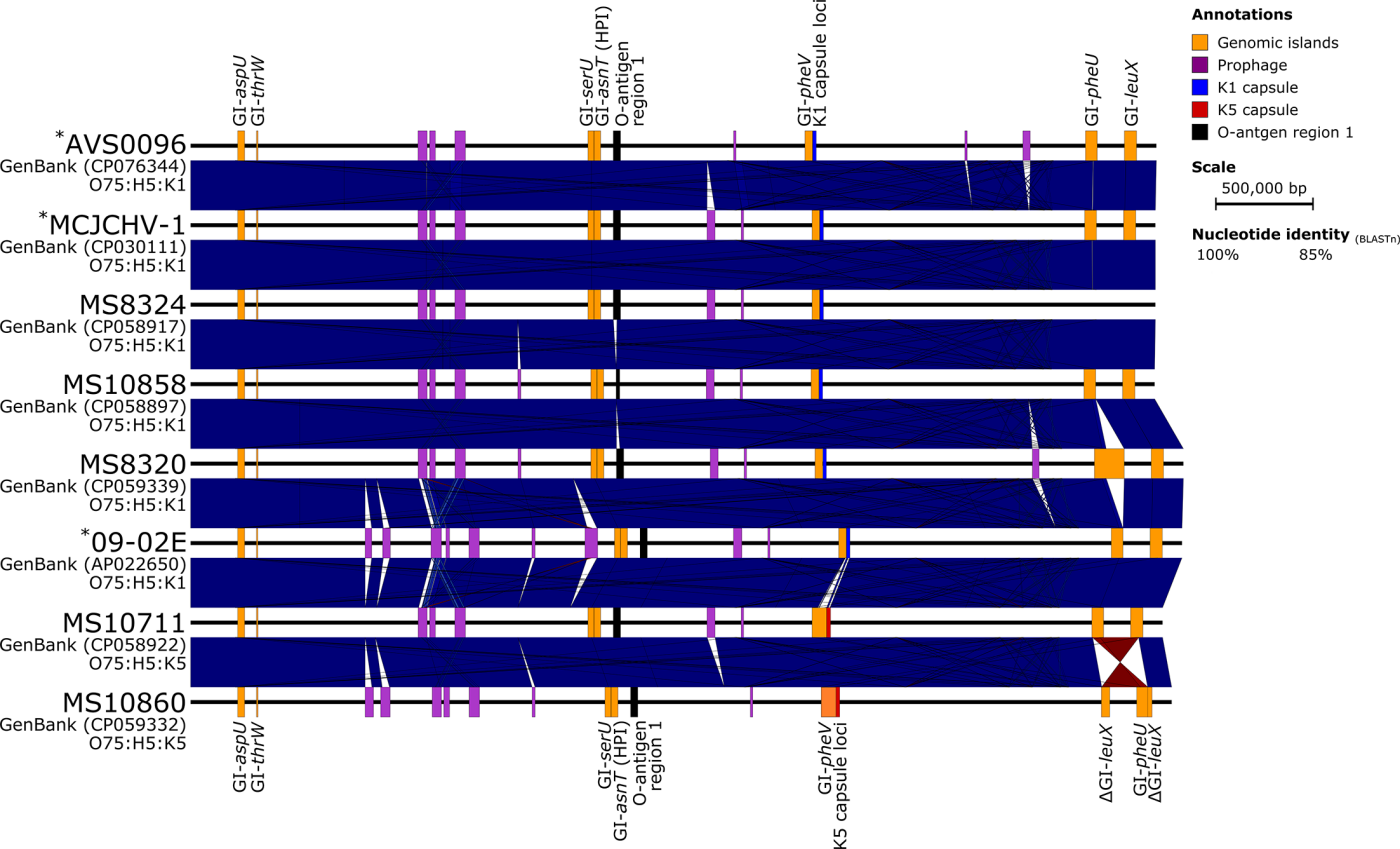
Whole-genome nucleotide pairwise comparisons between *Escherichia coli* sequence type (ST)1193 strains. Linear nucleotide alignment of the whole genome, highlighting chromosomal mobile genetic elements shared between genomes. Shared prophages and genomic islands (GIs) are shown in purple and orange, respectively. Blue and red (inversions) shading indicates nucleotide identity between sequences according to BLASTn (85–100%). Strains not sequenced in this study are indicated (*). Image created using Easyfig [80].

The complete genomes of five Australian ST1193 and three published completed ST1193 genomes are described here. *In silico* genotyping confirmed all eight strains as ST1193, with an O75:H5 serotype and *fim*H64 type 1 fimbrial adhesin, consistent with previous phenotypic characterizations of ST1193 [23,33]. Additionally, general features such as the number of predicted coding DNA sequences (CDS) and GC content were consistent across each strain and are listed in Table 1. The ST1193 strain MS10858 was selected as the reference genome for comparative analyses, as it represents the earliest reported clinical UTI isolate to date, collected in October 2007 (as of 21 October 2021). To characterize chromosomal features and identify differences, the chromosome of MS10858 underwent a BLASTn comparison against the remaining seven ST1193 complete genomes (Fig. 1), and nine other complete *E. coli* genomes chosen to represent different phylogroups and pathotypes (Fig. S1). A total of 33 regions of difference (RODs), between 1.9 and 62.6 kb in length, were defined in the chromosome of MS10858 (Fig. S1; Tables S2 and S3). These include seven genomic islands (GIs), four intact prophages and two partial prophages. The 20 remaining RODs primarily comprised genes associated with UPEC virulence. They included loci encoding flagella, O-antigen, the capsule, several putative fimbrial-like adhesins and a 1 947 bp GI-*argU* remnant. GIs were conserved across all eight ST1193 genomes (Fig. 1). However, some large insertions and translocations were present, including a 34 598 bp insertion in GI-*pheV* in MS10711. In MS10860, recombination between GI-*pheU* and GI-*leuX* resulted in a large (>100 kb) inversion of the intervening chromosome. Assembly error was ruled out by examining the read mapping in this region (Fig. S2). There was also a 92 421 bp insertion into GI-*pheU in* MS8320. This region starts after the frameshifted integrase gene (locus tag: MS8320_4488) and carried numerous hypothetical proteins (*n*=63) and a putative fimbriae locus (locus tags: MS8320_4505 and MS8320_4506). In addition, BLASTn showed that this ROD was present in multiple other species, including *Proteus mirabilis*, *Morganella morganii* and *Providencia stuartii* (Table S4).

**Table 1.** Genomic descriptions of complete *Escherichia coli* sequence type (ST)1193 representative genomes

	*Escherichia coli* ST1193 strain							
**Characteristics**	**MS10711**	**MS10858**	**MS10860**	**MS8320**	**MS8324**	MCJCHV-1*	09-02E*	AVS0096*
O- and H-genotype	O75:H5	O75:H5	O75:H5	O75:H5	O75:H5	O75:H5	O75:H5	O75:H5
Type 1 fimbriae	*fimH*64	*fimH*64	*fimH*64	*fimH*64	*fimH*64	*fimH*64	*fimH*64	*fimH*64
Capsule	K5	K1	K5	K1	K1	K1	K1	K1
Collection year	2010	2007	2007	2012	2011	2015	2018	2020
Location	Australia	Australia	Australia	Australia	Australia	USA	Vietnam	Switzerland
Host	Human	Human	Human	Human	Human	Human	Human	River water
Clinical presentation	Urinary tract infection	Urinary tract infection	Urinary tract infection	Urinary tract infection	Urinary tract infection	Neonatal meningitis	Healthy	–
Anatomical site	Urine	Urine	Urine	Urine	Urine	Cerebrospinal fluid	Faeces	–
Alternative ID	GNB 2889	QU054	QU059	CS12-NT018	CS12-WA103	NMEC-O75	–	–
BioSample	SAMN10141433	SAMN10141460	SAMN10141462	SAMN10141488	SAMN10141490	SAMN09462202	SAMD00198301	SAMN19493560
GenBank accession	CP058922 to CP058924	CP058897 to CP058900	CP059332 to CP059338	CP059339 to CP059342	CP058917 to CP058921	CP030111 to CP030116	AP022650 to AP022659	CP076344 to CP076347
SRA accession	SRR12008673	SRR12008672	SRR12008671	SRR12008670	SRR12008669	–	–	SRS9116829
Genome coverage	184×	276×	177×	97×	196×	320×	143×	125×
Chromosome length (bp)	4976 233	4935 580	5023 252	5082 313	4939 743	4939 457	5075 911	4944 762
Predicted CDS	4642	4604	4709	4758	4605	4848	4831	4645
GC content (%)	50.59	50.63	50.63	50.51	50.61	50.60	50.62	50.62
No. of tRNA genes	92	91	90	92	91	90	94	85
No. of rRNA operons	21	22	21	22	22	22	22	22
No. of plasmids	2	3	6	3	4	5	9	3
F-type plasmid	F-:A1:B20	F-:A1:B10	F :A1:B20	F-:A1:B10	F-:A1:B10FIIK:A-:B-	F-:A1:B10	F-:A1:B10	F-:A1:B10
IncX1	Not detected	Not detected	Not detected	Not detected	Not detected	Not detected	Present	Present
Colicinogenic plasmid	Col(BS512)	Col(BS512)	ColpVCCol(BS512)	Col(BS512)	Col(BS512)	ColpVCCol(BS512)	Col(MP18)Col(BS512)	Col(BS512)
Untypeable cryptic plasmids	0	1	3	1	1	2	5	0

*Not sequenced as part of this study.

### All eight ST1193 strains display multidrug resistance

The five Australian ST1193 strains were subjected to AST and exhibited resistance (based on CLSI clinical breakpoints) to ampicillin (beta-lactam) and ciprofloxacin (fluoroquinolone). They also showed variable resistance to antibiotics in the aminoglycoside, folate inhibitor, beta-lactam/beta-lactamase inhibitor and tetracycline classes (Table 2). Using long**-**read sequencing we were able to fully characterize the repertoire and genomic context of all antibiotic resistance genes in all eight ST1193 genomes, and correlate resistance genotype with observed phenotypic susceptibilities (Table 2). Antibiotic resistance genes were exclusively carried on F-type plasmids among our isolates (Fig. S3). However, no antibiotic resistance gene was conserved entirely across all eight strains, resulting in some variation in both plasmid structure and antibiotic resistance gene content (Figs S4 and S5). All eight strains also carried known chromosomal point mutations in genes encoding DNA gyrase (*gyr*A S83L and D87N), topoisomerase IV subunit A (*par*C S80I) and topoisomerase IV subunit B (*par*E L416F), conferring resistance to fluoroquinolones (MICs >2 mg l^−1^). Additional details and findings regarding the genomic context of these antibiotic resistance genes and plasmids are summarized in the Supplementary Materials.

**Table 2.** Resistant MIC breakpoints (µg ml^–1^) and associated acquired antibiotic resistance genes and/or chromosomal mutations

	Escherichia coli sequence type (ST1193) strain						
**Characteristic**	MS10711	MS10860	MS10858	MS8320	MS8324	MCJCHV-1*	AVS0096*†
Capsule (chromosome)	K5	K5	K1	K1	K1	K1	K1
Plasmid	pMS10711A	pMS10860A	pMS10858A	pMS8320A	pMS8324A	pNMEC-O75A	pAVS0096-a
GenBank accession	CP058923	CP059333	CP058898	CP059340	CP058918	CP030112	CP076345
Plasmid length (bp)	98 451	104 838	93 277	181 199	89 795	88,421	101 018
Plasmid multi-locus sequence type	F-:A1:B20	F-:A1:B20	F-:A1:B10	F-:A1:B10	F-:A1:B10	F-:A1:B10	F-:A1:B10
**Beta-lactams**							
Amoxicillin/clavulanic acid	nd	nd	nd	nd	nd	nd	21 mm (S)
Ampicillin	>16 (R)	>16 (R)	>16 (R)	>16 (R)	> 6 (R)	>16 (R)	6 mm (R)
Piperacillin/tazobactam	≤8 (S)	≤8 (S)	≤8 (S)	=128 (R)	≤8 (S)	nd	nd
Ampicillin/sulbactam	=16 (I)	>16 (R)	=16 (I)	>16 (R)	=16 (I)	nd	nd
Cefazolin	FAIL	=2 (S)	=4 (I)	>16 (R)	=8 (R)	=4 (I)	6 mm (R)
Cefepime	≤2 (S)	≤2 (S)	=4 (SDD)	=8 (SDD)	≤2 (S)	≤4 (SDD)	21 mm (I)
Cefotaxime	nd	nd	nd	nd	nd	nd	13 mm (R)
Ceftazidime	≤1 (S)	=2 (S)	≤1 (S)	=2 (S)	≤1 (S)	≤1 (S)	nd
Ceftazidime/avibactam	≤2 (No Intp)	≤2 (No Intp)	≤2 (No Intp)	≤2 (No Intp)	≤2 (No Intp)	nd	nd
Ceftriaxone	≤0.5 (S)	≤0.5 (S)	≤0.5 (S)	≤0.5 (S)	≤0.5 (S)	≤0.5 (S)	nd
Ceftolozane/tazobactam	≤1 (S)	=2 (S)	≤1 (S)	=2 (S)	≤1 (S)	nd	nd
Aztreonam	≤1 (S)	≤1 (S)	=4 (S)	≤1 (S)	≤1 (S)	nd	nd
Doripenem	≤0.5 (S)	≤0.5 (S)	≤0.5 (S)	=4(R)	=1(S)	nd	nd
Ertapenem	≤0.25 (S)	≤0.25 (S)	≤0.25 (S)	≤0.25 (S)	≤0.25 (S)	nd	nd
Imipenem	=2 (I)	≤1 (S)	≤1 (S)	≤1 (S)	≤1 (S)	≤0.5 (S)	nd
Meropenem	≤0.5 (S)	≤0.5 (S)	≤0.5 (S)	≤0.5 (S)	=2 (I)	nd	nd
Antibiotic resistance genes	*bla*_TEM-1B_	*bla*_TEM-1B_	*bla*_TEM-1B_	*bla*_TEM-1B_	*bla*_TEM-1B_	*bla*_TEM-1B_	*bla*_CTX-M-27_
**Fluoroquinolones**							
Ciprofloxacin	>2 (R)	>2 (R)	>2 (R)	>2 (R)	>2 (R)	>2 (R)	8 mm (R)
Levofloxacin	=8 (R)	=8 (R)	>8 (R)	=8 (R)	=8 (R)	nd	nd
Chromosomal point mutations	*gyrA* (S83L and D87N), *parC* (S80I) & *parE* (L416F)						
**Aminoglycosides**							
Tobramycin	>8 (R)	=8 (I)	=8 (I)	≤2 (S)	≤2 (S)	=8 (I)	nd
Amikacin	≤8 (S)	≤8 (S)	=16 (S)	≤8 (S)	≤8 (S)	≤8 (S)	nd
Gentamicin	>8 (R)	>8 (R)	>8 (R)	≤2 (S)	≤2 (S)	>8 (R)	20 mm (S)
Antibiotic resistance genes	*aac(3')-IId*	*aac(3')-IId*	*aac(3')-IId*	Undetected	Undetected	*aac(3')-IId*	Undetected
	*aadA5*	*aadA5*	*aadA5*	Undetected	*aadA5*	Undetected	*aadA5*
**Macrolides**							
Azithromycin	nd	nd	nd	nd	nd	nd	9 mm (R)
*Antibiotic resistance genes*	*mphA*	*mphA*	*mphA*	*mphA*	*mphA*	Undetected	*mphA*
**Tetracyclines**							
Tetracycline	≤4 (S)	>8 (R)	>8 (R)	≤4 (S)	≤4 (S)	≤4 (S)	nd
Tigecycline	≤1 (No Intp)	≤1 (No Intp)	≤1 (No Intp)	≤1 (No Intp)	=4 (No Intp)	≤1 (No Intp)	7 mm (R)
Antibiotic resistance genes	Undetected	*tetA*& *tetR*	*tetA*& *tetR*	Undetected	Undetected	Undetected	*tetA*& *tetR*
**Trimethoprim**							
Trimethoprim/sulfamethoxazole	>4 (R)	>4 (R)	>4 (R)	≤2 (S)	>4 (R)	≤2 (S)	6 mm (R)
Antibiotic resistance genes	*sul1*& *sul2*	*sul1*& *sul2*	*sul1*& *sul2*	Undetected	*sul1*	Undetected	*sul1*& *sul2*
	*drfA17*	*drfA17*	*drfA17*	Undetected	*drfA17*	*Undetected*	*drfA17*
**Other**							
Streptomycin	nd	nd	nd	nd	nd	nd	6 mm (R)
Antibiotic resistance genes	Undetected	*strA*& *strB*	*strA*& *strB*	Undetected	Undetected	*Undetected*	*strA*& *strB*
Chloramphenicol	nd	nd	nd	nd	nd	nd	23 mm (S)
Nitrofurantoin	≤32 (No Intp)	≤32 No Intp)	≤32 (No Intp)	≤32 (No Intp)	≥64 (No Intp)	nd	20 mm (S)

*Complete ST1193 genomes not sequenced in this study. Antibiotic resistance genes, Resfinder (updated 19 April 2020); (R) resistant; (I) intermediate; (SDD) susceptible dose dependent; (S) susceptible; (No Intp) no interpretation; (nd) not determined.

†Disc zone interpretive criteria.

### Key virulence-associated regions of difference are adjacent to major regions of recombination distribution

**Fig. 2.**
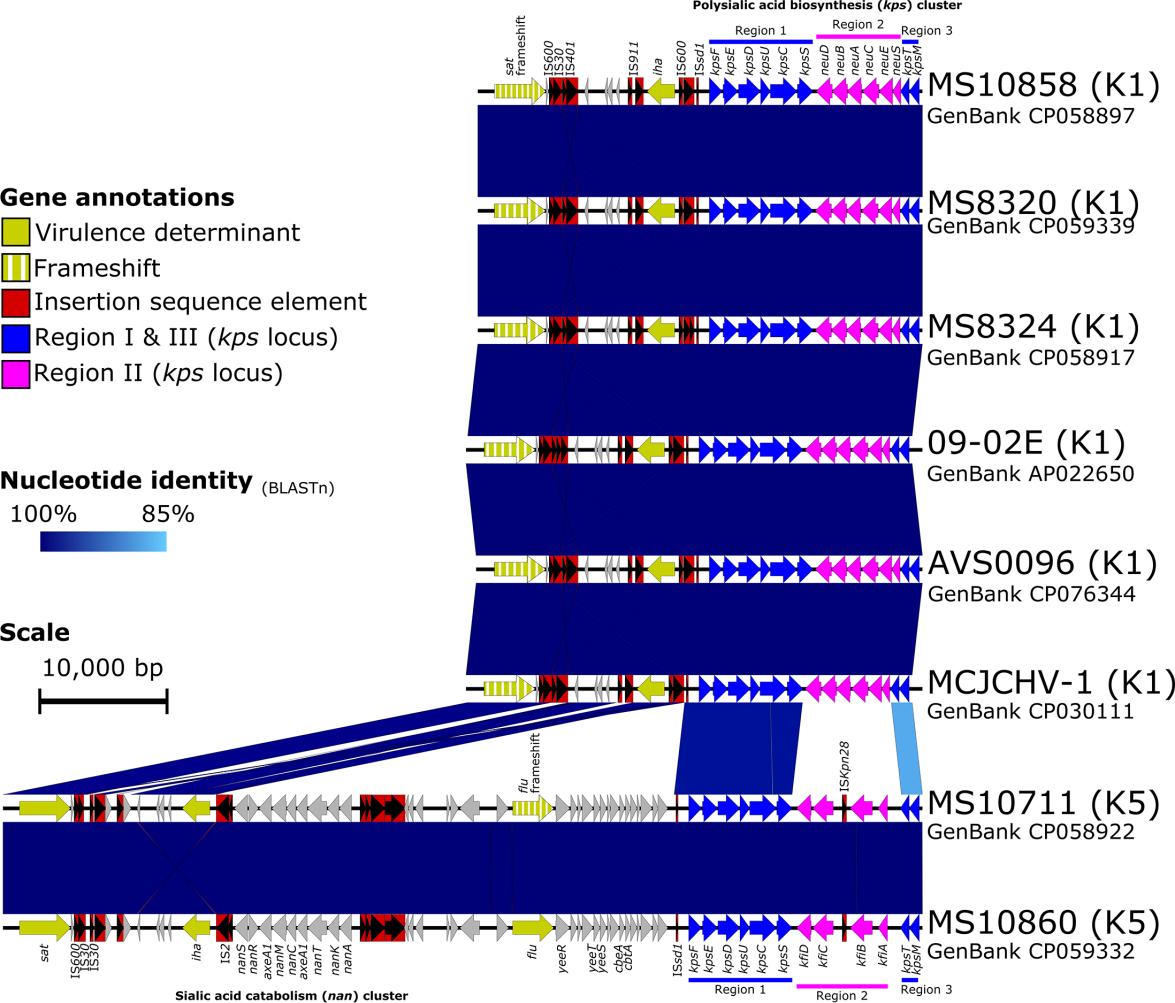
Linear nucleotide comparisons of the group 2 capsules in *Escherichia coli* sequence type (ST)1193 strains. Blue shading indicates nucleotide identity between sequences according to BLASTn. Key genomic regions are indicated: insertion sequences (IS): red; conserved capsular regions I and III: blue; differing capsular regions (region II): pink; virulence determinants: yellow; other CDS: grey. Image created using Easyfig [80].

The capsular locus was the most notable chromosomal difference between the eight ST1193 strains, aside from mobile genetic elements (Fig. 2). Specifically, changes in the polysialic acid biosynthesis (*kps*) cluster have probably resulted from a recombination-mediated switch from a K5 (MS10711 and MS10860) to a K1 (MS10858, MS8320 and MS8324) capsule [13]. To confirm this, the eight complete ST1193 chromosomes underwent a reference-free global alignment (5 399 477 bp), which was then used to predict regions of recombination using Gubbins. When using MS10858 as a reference, this identified several major regions of recombination with high SNP densities in and surrounding key regions associated with virulence, including GIs, prophages and the capsular locus (Fig. S6). Four regions of recombination between 9151 and 19 547 bp were found to encompass the GI-*pheV* and capsular locus, confirming that the capsular switch is probably the result of recombination.

Furthermore, iron acquisition is a critical feature of ST1193, evidenced by the presence of siderophores, along with various virulence genes associated with extra-intestinal pathogenic *E. coli* (ExPEC) virulence, as well as haem receptor and ferric citrate transporter systems found in Australian ST1193 chromosomes. All eight ST1193 genomes analysed in this study carried essential siderophores, such as enterobactin, yersiniabactin and aerobactin. These siderophores, along with the *iucABCD*/*iutA*, *sat* and *iha* loci, shared high nucleotide identity with virulence plasmids in other *E. coli* strains, further underlining their role in ExPEC virulence. Additionally, the Australian ST1193 chromosomes exhibited haem receptors encoded by the *chu* operon and the ferric citrate transporter system encoded by *fecIR* and *fecABCDE*, along with conserved principal iron/manganese transporters encoded by the *sitABCD* locus. Further details of the iron acquisition mechanisms can be found in the Supplementary Materials. Additional virulence genes, including those associated with iron acquisition, are summarized in Table S5.

We confirmed phenotypically that the Australian ST1193 strains have differing capsules (Fig. S7). K5 capsule encoding strains had an ~34.6 kb insertion downstream of GI-*pheV* that was absent in the K1 capsule encoding strains. This ~34.6 kb region carried the *flu* gene (encodes antigen 43) and a *nan*-operon (sialic acid catabolism), and is downstream of the *iha* adhesin (locus tags MS10711_3080 and MS10860_3153). Notably, the *flu* gene was intact in MS10860 (locus tag MS10860_3176) but was disrupted by frameshift mutations (possible pseudogene) in MS10711 (locus tags MS10711_3103 to MS10711_3106).

### Phylogenetic relationship of the complete ST1193 genomes

**Fig. 3.**
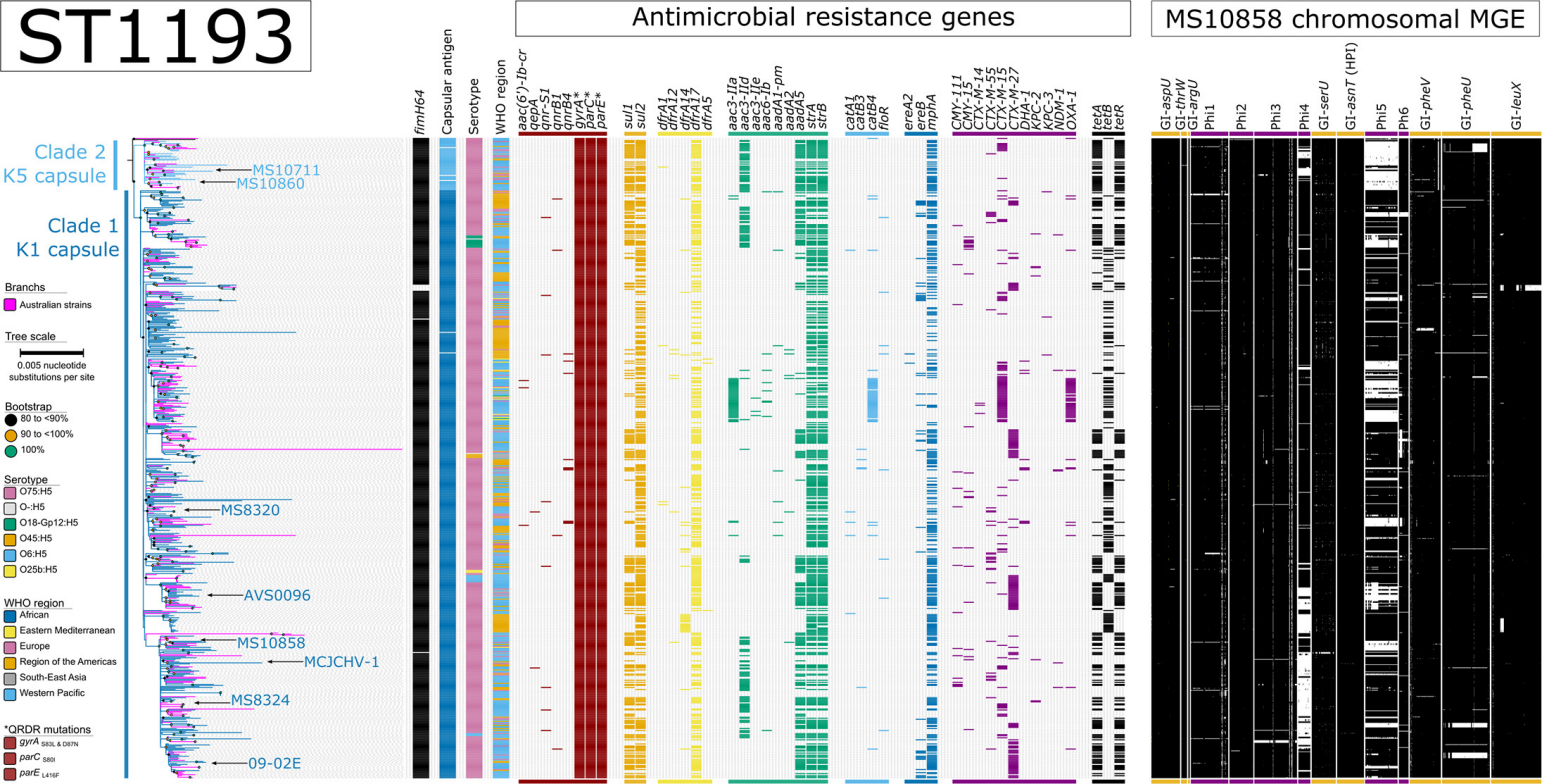
Maximum likelihood phylogeny of *Escherichia coli* sequence type (ST)1193 isolates, alongside the antimicrobial resistance genotype and mobile genetic element (MGE) complement. The phylogeny was inferred from 9687 non-recombinant orthologous biallelic core-genome SNPs from 624 strains. SNPs were derived from a core-genome alignment of 2461 300 bp and are called against the reference chromosome MS10858 (GenBank: CP058897). The phylogenetic tree is rooted according to the strain HICF686 (SRA: SRS1221162) outgroup, which has been omitted for visual purposes. Bootstrapping using 1000 replicates demonstrates the robustness of the branches. The presence/absence analysis of loci is based on the uniform coverage at each 100 bp window size in SPANDx. Coverage is shown as a heat map where ≥50% identity is highlighted in yellow and ≥80% identity is highlighted in black. White plots indicate regions with <50% coverage and may contain regions that are absent.

We revisited the population structure of ST1193 by incorporating the five Australian ST1193 genomes with a global ST1193 collection (*n*=619). By using the chromosome of MS10858 as a reference, the core-genome alignment between the 624 genomes comprised approximately 2 461 300 bp (based on regions estimated to the nearest 100 bp with ≥95% coverage across all genomes), which represents 49.9% of the MS10858 chromosome. A total of 14 327 orthologous, biallelic core-genome SNPs called against the reference chromosome MS10858 were identified. After removing 4640 SNPs within identified regions of recombination, an alignment of 9687 non-recombinant, orthologous, biallelic core-genome SNPs remained and was used to construct a maximum likelihood phylogenetic tree (Fig. 3).

This analysis of 624 ST1193 strains showed the lineage tightly clustered, with a median pairwise SNP distance of 55 SNPs [interquartile range (IQR): 44–69]. Notably, the maximum pairwise SNP distance was 314 SNPs between Australian strains AUSMDU00035640 (collected in 2019; SRA: SRS9466797) and AUSMDU00018204 (collected from human sputum in 2019; SRA: SRS8335820).

The ST1193 phylogeny resolved into two well-supported and diverse clades which have been named Clade 1 and Clade 2 (Fig. 3). Clade 1 was predominant (*n*=573/624 strains, 91.8%) amongst the global ST1193 population, whereas Clade 2 was less common (49/624 strains, 7.9%). The 573 strains in Clade 1 were collected across 30 countries spanning six WHO regions. Over 75% of the strains in Clade 1 were collected from Australia (*n*=200, 34.9%), the USA (*n*=127, 22.2%), Canada (*n*=37, 6.5%), Singapore (*n*=36, 6.3%) and China (*n*=31, 5.4%). The 49 strains within Clade 2 were collected across ten countries spanning four WHO regions. More than half of the strains within Clade 2 were collected in Australia (*n*=29/49, 59.2%). There was no discernible difference in host specificity between Clades 1 and 2. Clade 1 primarily comprised strains collected from humans (*n*=533/573, 93.0%) and domesticated dogs (*n*=18/573, 3.1%), with fewer strains from the environment (*n*=10/573 1.7%), silver gulls (*n*=7,573 10.2%) and food (*n*=4/573, 0.7%). Similarly, Clade 2 consisted primarily of strains from humans (*n*=42/49, 89.4%) and domesticated dogs (*n*=7/49, 14.9%). Of the 533 Clade 1 strains collected from humans, 314 have descriptive clinical sample metadata; 145 (46.2%) were from urine, 88 from blood (28.0%), 62 (19.7%) from faecal samples and 8 (2.5%) from sputum. Of the 42 Clade 2 strains collected from humans, 26 (53.1%) were collected from urine, three (6.1%) from faecal samples and a single strain from blood. The other 12 genomes were from the public domain and are missing sample type metadata.

Relative to MS10858, only five and eight SNPs defined the terminal branches to Clade 1 and Clade 2, respectively. Global strains within Clade 1 were separated by a maximum pairwise SNP distance of 314 SNPs (median: 54, IQR: 43–66). Meanwhile, global strains within Clade 2 were separated by a maximum pairwise SNP distance of 113 SNPs (median: 39, IQR: 27–50).

## Discussion

The fluoroquinolone-resistant ST1193 lineage has emerged worldwide as a significant extraintestinal pathogen [13]. The five Australian ST1193 strains sequenced in this study using long-read nanopore sequencing provide a broader representation of the known ST1193 major clades [13] and include the earliest known reported cases of clinical ST1193 involved in UTIs [19], thereby expanding on previous descriptions from incomplete draft assemblies [35]. Long-read sequencing highlighted (i) the low pairwise genomic diversity of strains from both Australia and globally, and (ii) the high conservation of the core and accessory genome (both chromosome and plasmid). Additionally, this study confirmed that the major differences in the capsular loci (K1 and K5) resulted from recombination, similar to previous reports [27,36]. These genomes also contained a similar F-type plasmid backbone, with six of the eight strains carrying a similar multidrug resistance region defined by either short or long variants of this resistance region (Fig. S5).

The inclusion of the reference genome for strain MS10858 provides crucial insight, representing one of the initial clinical isolates sequenced within the prevalent ‘K1-associated’ clade of ST1193 [19]. Contrasting the remaining seven complete ST1193 genomes with MS10858, we observed a remarkable level of genomic preservation, characterized by minimal pairwise SNP distances, except for localized regions of intense SNP clustering attributed to potential recombination events (Fig. S8). Analysis of the accessory genome also indicated a close evolutionary relationship, with four prophages and all GIs largely conserved across all strains (albeit with some large indels and translocations). Notably, while the prophages in MS10858 were specific primarily to our ST1193 collection, most GIs that encode virulence determinants have been identified in other ExPEC complete genomes. For example, the content and arrangement of modules in the MS10858 GI-*pheV* and GI-*leuX* were homologous with the ST131 reference strain EC958, suggesting that these regions are important to ST1193 fitness, as shown in ST131 [62]. A recent study has demonstrated that ST1193 emerged via simultaneous homologous recombinations in 11 gene loci from other *E. coli* [36]. This may be responsible for the high number of putative GIs in the chromosome of MS10858 seen in our results.

The collection of eight complete genomes now represents fully resolved capsular loci from both K5- and K1-associated ST1193. Our analysis showed that four regions of recombination between 9151 and 19 547 bp encompassed the GI-*pheV* and capsular locus, confirming that the capsular switch is probably the result of recombination. However, whether this was a single recombination event or multiple events remains to be elucidated. K1 and K5 capsule loci share conserved regions (regions I and III) that encode the transmembrane complex involved in the export and assembly of *E. coli* Group 2 capsular polysaccharides [40,63, 64]. However, region II is serotype-specific and encodes enzymes responsible for synthesizing the capsular polysaccharide. The K5 antigen is a heteropolymer of glucuronic acid and *N*-acetyl glucosamine [65], whereas the K1 antigen is a homopolymer of α2–8 linked *N*-acetylneuraminic acid (NeuNAc) [66,67], which is immunochemically identical to the group B *Neisseria meningitidis* capsular polysaccharide [68]. The K5 capsule, like many other K antigens, only provides protection to serum killing [69], whereas the K1 capsule also confers resistance to phagocytic killing [70]. Together, these observations indicate that *E. coli* presenting a K1 antigen can evade host immunogenic responses and may reach a threshold level of bacteraemia necessary for meningeal invasion [71], as was seen in the case of lethal neonatal meningoencephalitis, associated with O75:H5:K1-ST1193 strain MCJCHV-1 [21].

Outside of the ST1193 lineage, exchange of the *kps* locus has also been reported in the globally predominant *E. coli* ST131 lineage [72]. The switching of region II in the *kps* locus is described as the predominant mechanism of K-antigen-type sharing between genome pairs belonging to different clonal lineages [73]. Our findings concerning the role of recombination imply that the capsule locus in ST1193 is under selective pressure, where the acquisition of a capsular locus with a different *kps* region II leading to the switch between K5 and K1 antigen (thus increasing in capacity to evade host immunogenic responses) may have primed the ST1193 lineage for its recent population expansion [13]. A similar capsule locus switching was associated with the emergence of vaccine escape variants and subsequent population expansions in *Streptococcus pneumoniae* [74]. However, no evidence of a ‘straightforward relationship’ between the *kps* locus switching and different lineages was detected when investigating population frequency and locus gene content [73].

The availability of eight complete ST1193 genomes allowed us to extend previous ST1193 studies that targeted either a specific repertoire of genes, or were unable to fully resolve the surrounding genomic context of key virulence and resistance genes [26,33]. For example, we showed that the yersiniabactin and aerobactin siderophore loci were carried on mobile genetic elements in ST1193. ST1193 also contained the enterobactin siderophore, like that present in the ST131 strain EC958. The conservation of this siderophore in other UPEC lineages indicates its importance in the fitness of ST1193. This has been exemplified in the work by Pi *et al.* [75], demonstrating the essential function of enterobactin secretion and transportation in colonizing the healthy murine gastrointestinal tract by *E. coli.* When compared to the importance of enterobactin secretion and transportation within the host [75], an increased siderophore repertoire (such as the yersiniabactin and aerobactin loci in ST1193) carried on mobile genetic elements may have been more important in the evolution and dissemination of ST1193, as these strains are more likely to outcompete other bacteria in sequestering iron in the densely populated gastrointestinal tract [76,77]. Notably, an earlier study that screened ST1193 isolates from a Chinese hospital between 2014 and 2015 reported the genes *iutA* and *fyuA* (carried on the aerobactin and yersiniabactin loci, respectively) at a frequency of over 90% [26].

AST of these eight ST1193 strains showed multidrug resistance, including resistance to critically important antibiotics like fluoroquinolones and cephalosporins. In most cases, the phenotype matched the genotype, but strain MS8320 also showed *in vitro* resistance to piperacillin/tazobactam, ampicillin/sulbactam, cefazolin and doripenem (carbapenem), suggesting that eight copies of the *bla*_TEM-1B_ gene carried on IS*26*-mediated transposable units in a tandem array confer additional resistance phenotypes. A similar mechanism of piperacillin/tazobactam resistance due to IS*26*-mediated amplification of *bla*_TEM-1B_ in *E. coli* has previously been reported [78]. Similarly, tandem arrays which increase the copy number of genes encoding resistance to beta-lactams have been shown to confer resistance to carbapenems [79]. Fluoroquinolone resistance was attributed to vertically transmitted point mutations within the quinolone resistance-determining regions of *gyrA*, *parC* and *parE*, like other UPEC lineages. However, unlike the stepwise acquisition of mutations conferring fluoroquinolone resistance in the *E. coli* ST131 lineage, resistance to fluoroquinolones in ST1193 results from multiple recombination events [36]. Six of the eight ST1193 strains (excluding MCJCHV-1 and MS8320) also featured a similar resistance region on a homologous F-type plasmid, differing primarily on whether the short or long variant was present and the arrangement of gene modules. Although we cannot be sure of the events leading to the evolution of these plasmids, it is notable that variants of this resistance region featured in strains across both major clades of the ST1193 lineage.

The mutations in DNA gyrase and topoisomerase genes that encode resistance to fluoroquinolones were not present in non-ST1193 isolates within the basal CC14 lineages [13], except for the outgroup strain HICF686 (ST6460; K5; SRA: SRS1221162), which contains a single mutation in *gyrA* (S83L). Notably, the type 1 fimbrial adhesin was defined as type *fimH*64, which has been previously reported as a single-locus variant of *fimH*27 [C314T (P125L)] relative to other CC14 strains such as HICF686 [23]. Of most interest and supported by Johnson *et al*. [13], the capsular locus in ST14 sub-lineages is K5. This indicates that the ancestral capsule type in the ST1193 lineage is K5 and that a switch to K1 has occurred prior to the expansion of the ‘K1-associated’ ST1193 clade. A defining characteristic of Clade 1 strains is carriage of the K1 capsular antigen (referred to as the ‘K1-associated’ lineage by Johnson *et al*. [13]), in contrast to carriage of the K5 capsular antigen in Clade 2 strains (referred to as the ‘K5-associated’ lineage [13]).

This high-resolution study in the genomics of ST1193 had two main strengths. The first was the contribution of three additional complete reference genomes representing strains from the major ‘K1-associated’ lineage, and two genomes from the ‘K5-associated’ lineage. The addition of these five strains with the three already available complete reference genomes of ‘K1-associated’ strains will help improve our knowledge of the pathobiology of ST1193. The second strength was that long-read sequencing enabled in-depth analysis of the core and accessory genome. This allowed for confirmation of previous assertions regarding capsule switching due to recombination. Long-read sequencing also allowed high-resolution examination of both antibiotic-resistance gene context and copy number, which was important for predicting antibiotic resistance phenotypes from the genotype. However, sequencing only five genomes to completion was a limitation in this study. As such, we acknowledge that phylogenetic inference on this small sample size alone would inappropriately underestimate the overall mutations across the ST1193 population. However, the availability of eight publicly available and published genomes (as of 2021) that are representative of the major ‘K1-associated’ and ‘K5-associated’ ST1193 lineages now offers opportunities for further large-scale phylogenetic analyses using more appropriate, closely related reference genomes. A larger sample size is critical for further understanding of transmission, adaptation and the evolution of ST1193.

## Conclusion

We used ONT and Illumina sequencing to complete the first five representative ST1193 genomes from patients with UTIs in Australia. These genomes represent high-quality, circularized, complete reference genomes for future comparative genomics studies of ST1193. Here, we expanded on previous studies of ST1193 by providing the genomic context of virulence and antibiotic resistance gene content and highlighted the important role of mobile genetic elements in shaping this lineage. While ST1193 represents a new threat to global human health, its emergence and spread as a fluoroquinolone-resistant clone appears to be driven by different mechanisms than ST131, including the acquisition of fluoroquinolone resistance due to recombination prior to a switch from the ancestral K5 (CC14 lineage) to K1 capsule type, and the acquisition of numerous mobile genetic element-borne virulence-associated siderophore loci. Detailed ongoing analyses of ST1193, and the incorporation of detailed epidemiological data, may help us to pinpoint triggers that further define the successful global expansion of this lineage and allow us to predict the emergence of new resistant clones in the future.

## supplementary material

10.1099/acmi.0.000894.v3 Uncited Fig. S1.

10.1099/acmi.0.000894.v3 Uncited Table S1.

## Abbreviations

AGAR: Australian Group on Antimicrobial Resistance
ARC: Australian Research Council
AST: antibiotic susceptibility testing
BMD: broth microdilution method
CC: clonal complex
CDS: coding DNA sequence
CLSI: Clinical and Laboratory Standards Institute
EUCAST: European Committee on Antimicrobial Susceptibility Testing
ExPEC: extra-intestinal pathogenic *Escherichia coli*
GI: genomic island
GTR: general time reversible
IQR: interquartile range
IS: insertion sequence
LB: lysogeny broth
NCBI: National Center for Biotechnology Information
NCRIS: National Collaborative Research Infrastructure Strategy
NHMRC: National Health and Medical Research Council
ONT: Oxford Nanopore Technologies
QFAB: Queensland Facility for Advanced Bioinformatics
ROD: region of difference
SIR: sensitive, intermediate and resistant
SNP: single nucleotide polymorphism
SRA: sequence read archive
ST: sequence type
UPEC: uropathogenic *Escherichia coli*
UQCCR: University of Queensland Centre for Clinical Research
UTI: urinary tract infection
WGS: whole-genome sequencing
WHO: World Health Organization
