# Supplementary material for: The complete genome sequence of five pre-2013 Escherichia coli sequence type (ST)1193 strains reveals insights into an emerging pathogen
Source: Access Microbiol. 2024 Oct 18;6(10):000894.v3. doi: 10.1099/acmi.0.000894.v3 (PMC11488385; doi:10.1099/acmi.0.000894.v3)
Supplement: Uncited Fig. S1. [file acmi-6-00894-s001.pdf]

The complete genome sequence of five historical *Escherichia coli* sequence type (ST)1193 strains reveals insights into an emerging pathogen

1.1 Author names

Rhys T. White[1,2,3*], Melinda M. Ashcroft[1,2], Michelle J. Bauer[4], Jan Bell[5], Dominika Butkiewicz[4], Laura Álvarez-Fraga[1,6], Justine S. Gibson[7], Amanda K. Kidsley[5], Joanne L. Mollinger[8], Kate M. Peters[1,9], Minh-Duy Phan[1,9], Leah W. Roberts[1,2,4], Benjamin A. Rogers[10], Mark A. Schembri[1,9,11], Darren J. Trott[5], John Turnidge[7], Brian M. Forde[9,11#], Scott A. Beatson[1,2,11#]

1.2 Affiliation(s)

[1]The University of Queensland, School of Chemistry and Molecular Biosciences, Brisbane, Queensland 4072, Australia

[2]The University of Queensland, Australian Centre for Ecogenomics, Brisbane, Queensland 4072, Australia

[3]Institute of Environmental Science and Research, Health Group, Porirua 5022, New Zealand

[4]The University of Queensland, UQ Centre for Clinical Research (UQCCR), Royal Brisbane & Women's Hospital Campus, Herston, Queensland 4029, Australia

[5]The University of Adelaide, School of Animal and Veterinary Sciences, Roseworthy, South Australia 5371, Australia

[6]University of Montpellier, INRAE, Le Laboratoire de Biotechnologie de l'Environnement, Narbonne 11100, France

[7]The University of Queensland, School of Veterinary Science, Gatton, Queensland 4343, Australia

[8]Biosecurity Sciences Laboratory, Biosecurity Queensland, Department of Agriculture and Fisheries, Coopers Plains, Queensland 4108, Australia

[9]The University of Queensland, Institute for Molecular Bioscience, Brisbane, Queensland 4072, Australia

[10]School of Clinical Sciences, Monash Medical Centre, Monash University, and Monash Infectious Diseases, Monash Health, Clayton, Victoria 3168, Australia

[11]The University of Queensland, Australian Infectious Disease Research Centre, Brisbane, Queensland 4072, Australia

#: These authors have contributed equally.

1.3 Corresponding author and email address

*Corresponding author: Rhys White; Telephone: +64-4-914-0700; E-mail: rhys.white@esr.cri.nz
Scott A. Beatson; Telephone: +61-7-33654863; Email: s.beatson@uq.edu.au

1.4 Keywords

Antibiotic resistance; capsule; phylogenetic analysis; long-read sequencing; mobile genetic elements

<u>This file includes the following:</u>

Supplementary Methods.

Supplementary Results.

Supplementary Figure S1. Genomic comparisons between the *Escherichia coli* MS10858 chromosome and other complete *E. coli* genomes.

Supplementary Figure S2. Alignment of Oxford Nanopore Technologies and Illumina sequence reads to the annotated sub-region of *Escherichia coli* sequence type (ST)1193 strain MS10860 that contains an inversion between the two GI-*leuX* fragments.

Supplementary Figure S3. Whole-genome nucleotide pairwise comparisons between *Escherichia coli* sequence type (ST)1193 F-type plasmids.

Supplementary Figure S4. Linear schematics of the antimicrobial resistance gene region carried on F-type plasmids in the eight complete *Escherichia coli* ST1193 strains.

Supplementary Figure S5. Linear nucleotide pairwise comparisons between two *Escherichia coli* sequence type (ST)1193 F-type plasmids highlighting resistance region variants.

Supplementary Figure S6. Genome-wide single-nucleotide polymorphism distribution against *Escherichia coli* strain MS10858.

Supplementary Figure S7. Cross-brush agar test results show K1 phage reaction with strain MS10858 and K5 phage reaction with strain MS10860.

Supplementary Figure S8. Distribution of single-nucleotide polymorphisms (SNPs) across the genome of *Escherichia coli* ST1193 strain MS10858 compared to the genomes of sequence type (ST)1193 strains MS10711, MS10860, MS8320, MS8324, MCJCHV-1, 09-02E, and AVS0096.

2. Supplementary Methods

2.1 Quality control for the Illumina sequence data

Raw reads were checked for quality using FastQC v0.11.9 (http://www.bioinformatics.babraham.ac.uk/projects/fastqc/, accessed 04 April 2024). To perform taxonomic profiling and detect *Escherichia coli* in the raw sequence data, we used Kraken v2.0.7-beta [1] with default parameters and an NCBI Reference Sequence (RefSeq) database [2], Standard (https://benlangmead.github.io/aws-indexes/k2, accessed on 05 April 2024). As previously described [3], Trimmomatic v0.36 [4] was used in paired-end mode to quality filter the raw reads by removing low-quality bases and read-pairs together with Illumina adaptor sequences (see Supplementary Materials, Table S6 for sequencing quality metrics).

2.2 Quality control for the nanopore sequence data

To create summary metrics, the Oxford Nanopore Technologies (ONT) sequence read data, in (compressed) FASTQ format, was input into NanoPlot v1.26.1 [5] with default parameters (see Supplementary Materials, Table S7 for sequencing quality metrics). Porechop v0.2.3 (https://github.com/rrwick/Porechop, accessed 05 April 2024) trimmed the adapter sequences from the raw reads, and the jsa.np.filter option in Japsa (https://github.com/mdcao/japsa/, accessed 05 April 2024) was used to remove reads with a sequence length below 300 bp and an average quality score below 10.

2.3 *de novo* assembly of the nanopore sequence read data

The quality-filtered long-read sequencing data were *de novo* assembled using Flye v2.5 [6, 7] with the '--plasmids' flag (to rescue short unassembled plasmids), an estimated genome size of 5 Mb, and otherwise default parameters. Each chromosome or plasmid was circularised by removing overlapping regions, which were identified by performing self-comparative BLASTn v2.9.0+ [8] searches (≥95% nucleotide identity) between the ends of the single contig before visualising using the Artemis Comparison Tool v18.1.0 [9]. Each chromosome and plasmid assembly then underwent five rounds of additional polishing by mapping the corresponding, filtered Illumina reads to each contig using BWA-MEM [10] and then correcting single nucleotide polymorphisms (SNPs) and small insertions and deletions (INDELs) with Pilon v1.23 [11].

3. Supplementary Results

3.1 Whole-chromosomal alignments reveal a highly conserved genome

Individual whole-chromosome alignments of the seven ST1193 strains against the MS10858 reference chromosome revealed pairwise SNP differences ranging from 69 (MCJCHV-1) to 915 (09-02E) (Supplementary Materials, Figure S8). Additionally, several mobile genetic elements were associated with a higher SNP density than the rest of the chromosome, consistent with the overestimation of SNPs in dynamic regions of the chromosome where indels and insertion sequences (IS) (repeat regions) predominate (Supplementary Materials, Figure S8) Similarly, the high SNP density within the capsule region of MS10711 and MS10860 is expected when comparing K5 capsular strains with the K1 capsular strain MS10858. When excluding SNPs within the capsule locus, prophages, or genomic islands (GIs), the pairwise SNP distances between MS10858 and the other ST1193 genomes in this study ranged from 63 to 349 SNPs, broadly consistent with pairwise SNP distances reported in *E. coli* lineages elsewhere [12].

3.2 Iron acquisition is a critical feature of ST1193

All eight ST1193 genomes contained the siderophores enterobactin (*entSABECFD*), yersiniabactin, and aerobactin (Supplementary Materials Table S5). The yersiniabactin locus was carried on the high-pathogenicity island (HPI; integrated into the tRNA-*asnT*), while the aerobactin locus was carried on GI-*pheV*. This shared *iucABCD*/*iutA*, *sat,* and *iha* locus has also been reported in virulence plasmids, is associated with extra-intestinal pathogenic *E. coli* (ExPEC) virulence [13], and has >99.8% nucleotide identity and 100% query coverage to corresponding loci in other *E. coli* strains. This provides further support in the role for the *iucABCD*/*iutA*, *sat*, and *iha* loci contributing to ExPEC virulence. Additionally, all five Australian ST1193 chromosomes carried a heme receptor (encoded by the chu operon) and the ferric citrate transporter system, which was encoded by *fecIR* and *fecABCDE* [14-17]. In our five Australian ST1193 genomes, the *fecIR* and *fecABCDE* operon was located on a 62.5 Kb GI integrated into the tRNA-*leuX*. The principal iron/manganese transporters (encoded by the *sitABCD* locus [18]) was conserved in all five ST1193 strains on a partial 29.7 Kb prophage that was most similar (n = 23/31 open reading frames with 100% nucleotide identity) to the *Escherichia* phage Lambda (GenBank: J02459).

3.3 Long-read sequencing revealed the genomic context of antimicrobial resistance genes

F-type plasmids are a common feature of uropathogenic *E. coli* (UPEC) and frequently carry an array of antibiotic resistance genes [19]. In all eight ST1193 complete genomes, acquired antibiotic resistance genes were carried on an F-type plasmid with a similar plasmid backbone (Supplementary Materials, Figure S3). *In silico* plasmid multi-locus sequence typing (MLST) determined that the plasmid type in the two ST1193 genomes encoding a K5 capsule (MS10711 and MS1860) was F-:A1:B20. However, in the remaining six genomes encoding a K1 capsule, the plasmid type was F-:A1:B10, as per previous reports [20]. In addition to a different IncFIB replicon, plasmids pMS10860A and pMS10711A differed from the other six plasmids by an approximate ~18.5 Kb region downstream of *repB*. This region was punctuated by IS and carried the *mmuMP* operon (S-Methylmethionine metabolism) and a Type I Restriction Modification System (RMS) with the specificity subunit matching 100% amino acid identity and coverage to S.Eco067II (GenBank: AXZ83755) of the *E. coli* ST405 strain AR_0067, which targets the palindromic sequence: 5'- CA^{6m}AYN$_6$C<u>T</u>GG-3' [21].

In all eight F-type plasmids, the region carrying antibiotic resistance genes started downstream of the *pemIK* toxin-antitoxin operon and except for pMS10858A ended upstream of *resA* (thiol-disulfide oxidoreductase ResA). This resistance region was smallest in pNMEC-O75A (10,110 bp) and carried a truncated Tn*2* element carrying *bla*$_{\text{TEM-1B}}$ (narrow-spectrum cephalosporin resistance). Immediately downstream was an IS*15*-flanked composite transposon carrying *aac(3')-IId* (aminoglycoside resistance) and a truncated IS*Kpn72*, with a truncated IS*Cfr1* and an IS*15* further downstream (Supplementary Materials, Figure S4A). Conversely, the largest resistance region was in pMS8320A, which carried a 106,799 bp composite transposon (referred to as Tn*7394* from now on), which was flanked by an IS*1A* in direct orientation and a truncated IS*1R* in the reverse orientation (Supplementary Materials, Figure S4B). The absence of Tn*7394* flanking repeats suggests no evidence of recent transposition. Tn*7394* carried a truncated Tn*As3* carrying a partial transposase and the *merTPCA* operon (mercury resistance). Following this was eight copies of an 11,630 bp IS*26* transposable unit (TU), which were arranged in a tandem array, with each adjacent transposon sharing a flanking IS*26*, as previously described [22]. This IS*26* TU comprised an IS*26*, a partial Tn*As3*, a second IS*26* in the reverse orientation, a truncated IS*6100*, *mphA* and a truncated Tn*2* carrying *bla*$_{\text{TEM-1B}}$. Notably, antibiotic susceptibility testing showed that MS8320 was also resistant to piperacillin/tazobactam, ampicillin/sulbactam, and cefazolin

and was the only ST1193 strain in this study to show resistance to doripenem (carbapenem), suggesting that multiple copies of *bla*$_{TEM-1B}$ confers additional resistance phenotypes.

Plasmids pAVS0096A and pA1_09_02E carried *bla*$_{CTX-M-27}$ downstream of the *pemIK* operon, which formed an IS*903B*-*bla*$_{CTX-M-27}$-ΔIS*Ecp1*-IS*15DIV* module (reversed in pA1_09_02E). In pMS1086A, pMS8624A and pMS10711A, however, the region downstream of *pemIK* carried a truncated Tn*2* carrying *bla*$_{TEM-1B}$ with an IS*15* immediately downstream. These five plasmids carried a similar resistance region downstream of their respective beta-lactamase modules (Supplementary Materials, Figure S4C). There were two major variants of this resistance region (Supplementary Materials, Figure S5). The short variant (found in pMS8324A and pMS10711A (reverse orientation)) started at an IS*26* and carried *mphA* (macrolide resistance), with *sul1, aadA5* (aminoglycoside resistance), and *dfrA17* (trimethoprim resistance) further downstream, flanked by an IS*6100* and an IS*15*. In addition to these antibiotic resistance genes, the long variant (found in pAVS0096A, pA1_09_08E and pMS10860A) carried a Tn*As3* fragment, *tetA* and *tetR* (tetracycline resistance), *aph(6')-ld* (also known as *strA*) and *aph(3'')-lb* (also known as *strB*) (sulphonamide resistance) and *sul2* (sulphonamide resistance) upstream of the IS*26*. Lastly, present in the strains pMS10860A, pMS10711A and pMS10858A was an IS*15* composite transposon carrying *aac(3')-IId* (aminoglycoside resistance), a truncated IS*Kpn72* and truncated IS*10R* (region reversed in pMS10711A). Plasmids pMS10860A and pMS10711A shared an additional antibiotic resistance genes module, immediately downstream of *bla*$_{TEM-1B}$ carrying IS*15*-hypothetical protein-*aac(3')-IId* (aminoglycoside resistance)-ΔIS*Kpn72*-ΔIS*10R*-IS*15*-ΔTn*As3*. Notably, the large resistance island variant was most like (>99.7% nucleotide identity and 100% sequence coverage) the resistance island in plasmid pSCU-147-1 (GenBank: CP054326) from the *E. coli* ST1193 strain SCU-147 (GenBank: CP054325), isolated from a rectal swab from a healthy college student in the United States in 2013 [23]. Lastly, plasmids pMS10860A and pMS10711A shared an additional antibiotic resistance genes module, immediately downstream of *bla*$_{TEM-1B}$ carrying IS*15*-hypothetical protein-*aac(3')-IId* (aminoglycoside resistance)-ΔIS*Kpn72*-ΔIS*10R*-IS*15*-ΔTn*As3*.

4. Supplementary Figures

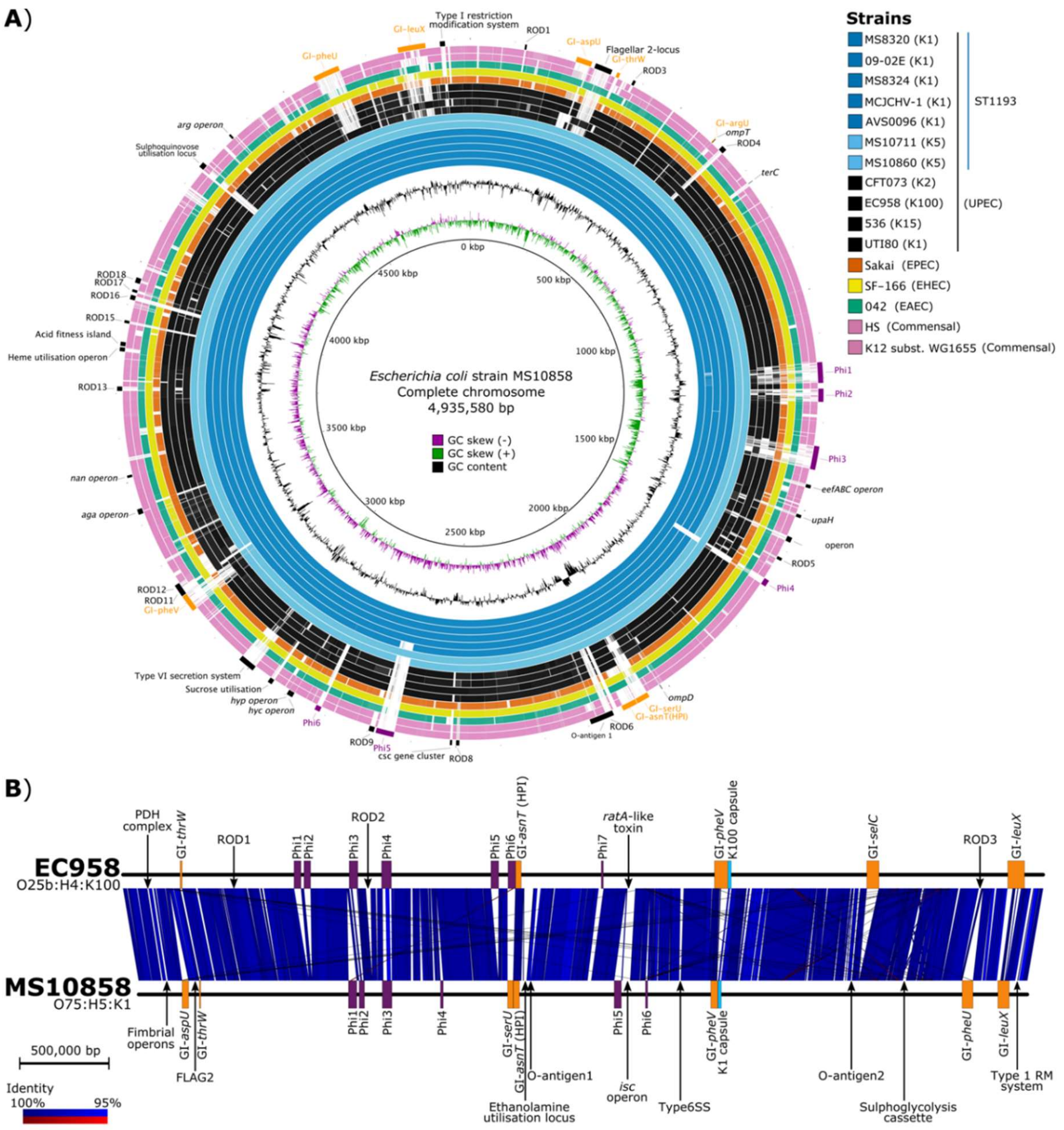

Supplementary Figure S1. Genomic comparisons between the *Escherichia coli* MS10858 chromosome and other complete *E. coli* genomes. (A) Circular representation of the *E. coli* MS10858 chromosome. The three innermost circles represent chromosome coordinates relative to MS10858, GC skew, and GC content. The degree of coloured shading indicates nucleotide identity between MS10858 and each *E. coli* chromosome (Rings 4 to 19). Key is ordered with the innermost ring at the top, descending to the outermost ring. Colours correspond to the *E. coli* pathotype, as shown in the legend. The outermost ring describes the positions of regions of difference (RODs; black), genomic islands (orange), and prophages (purple). Image created using BRIG [24]. (B) Linear nucleotide alignment of ST131 strain EC958 against ST1193 strain MS10858, highlighting mobile genetic elements and RODs between the two chromosomes. Blue and red (inversions) shading indicate nucleotide identity between sequences according to BLASTn (95 to 100%). Image created using Easyfig [25].

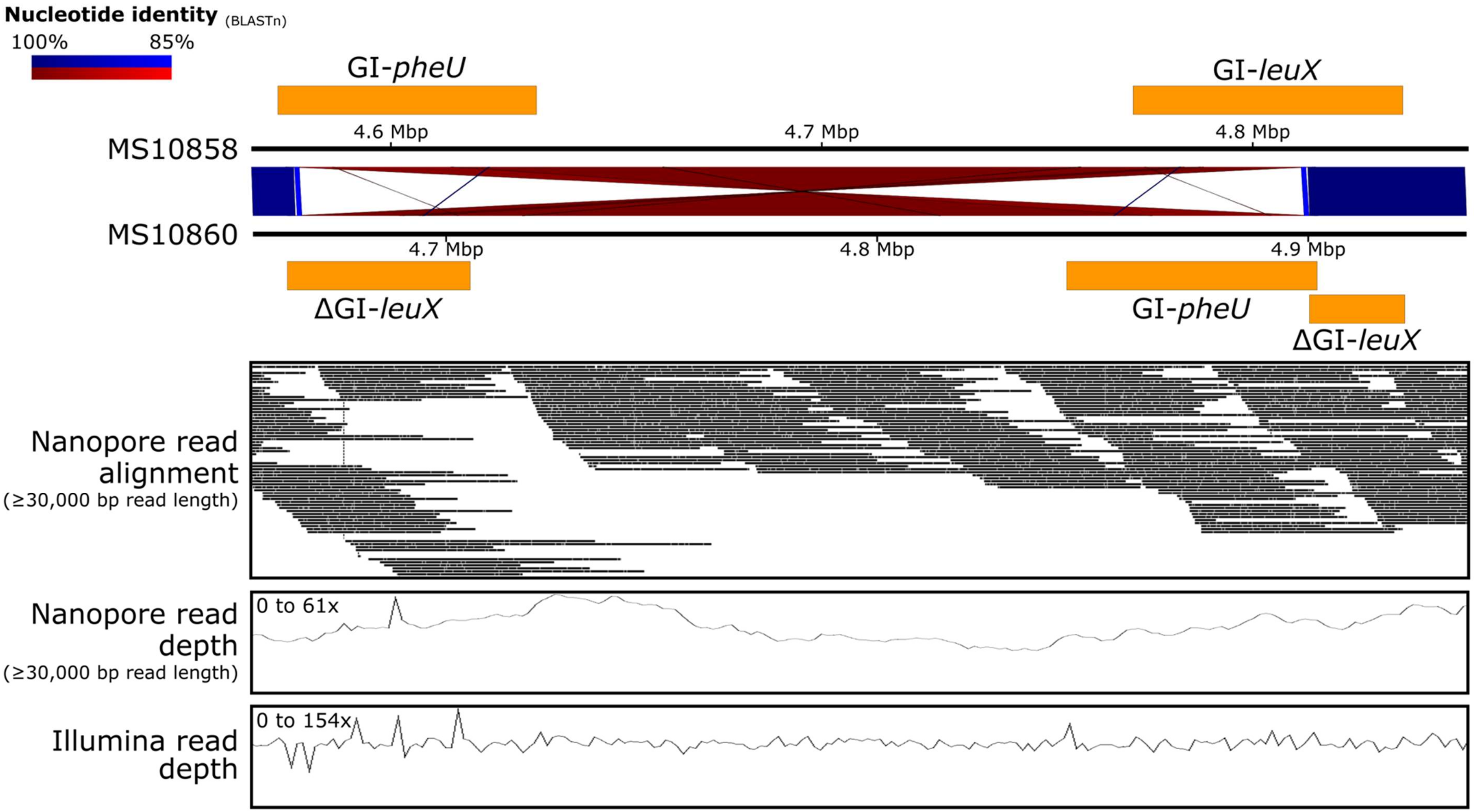

Supplementary Figure S2. Alignment of Nanopore and Illumina reads to the annotated sub-region of *Escherichia coli* sequence type (ST)1193 strain MS10860 that contains an inversion between the two GI-*leuX* fragments. Linear nucleotide alignment between strain MS10858 (p.4567620..4849976) and MS10860 (p.4655220..4936746). Blue and red (inversions) shading indicate nucleotide identity between sequences according to BLASTn (85 to 100%). Read mapping highlights the accuracy of the genome assembly.

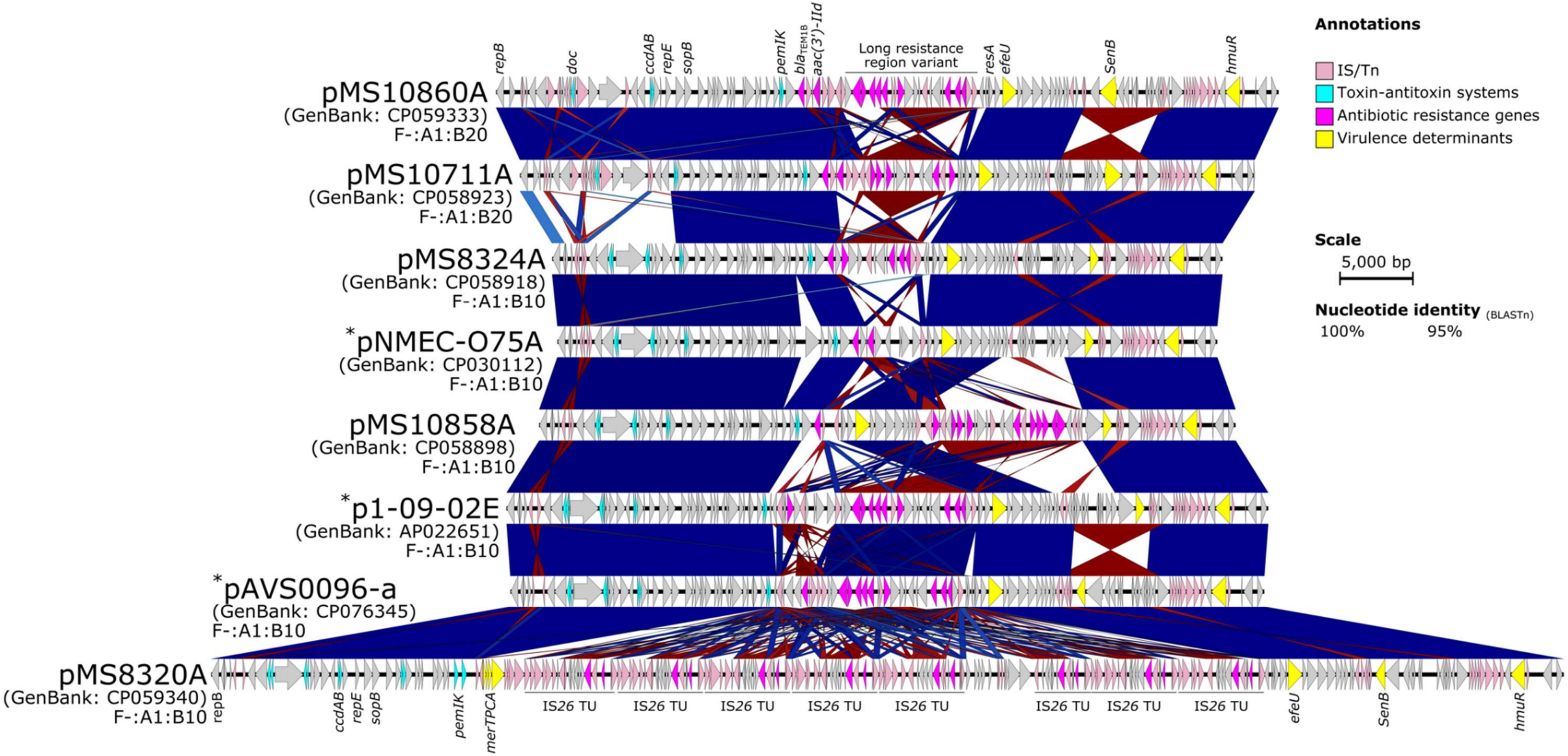

Supplementary Figure S3. Whole-genome nucleotide pairwise comparisons between *Escherichia coli* sequence type (ST)1193 F-type plasmids. Linear nucleotide alignment of the whole plasmid genome, highlighting mobile genetic elements shared between genomes. Blue and red (inversions) shading indicate nucleotide identity between sequences according to BLASTn (95 to 100%). Antimicrobial resistance and virulence genes are labelled and coloured pink and yellow, respectively. Insertion sequence elements are also coloured red. Image created using Easyfig [25].

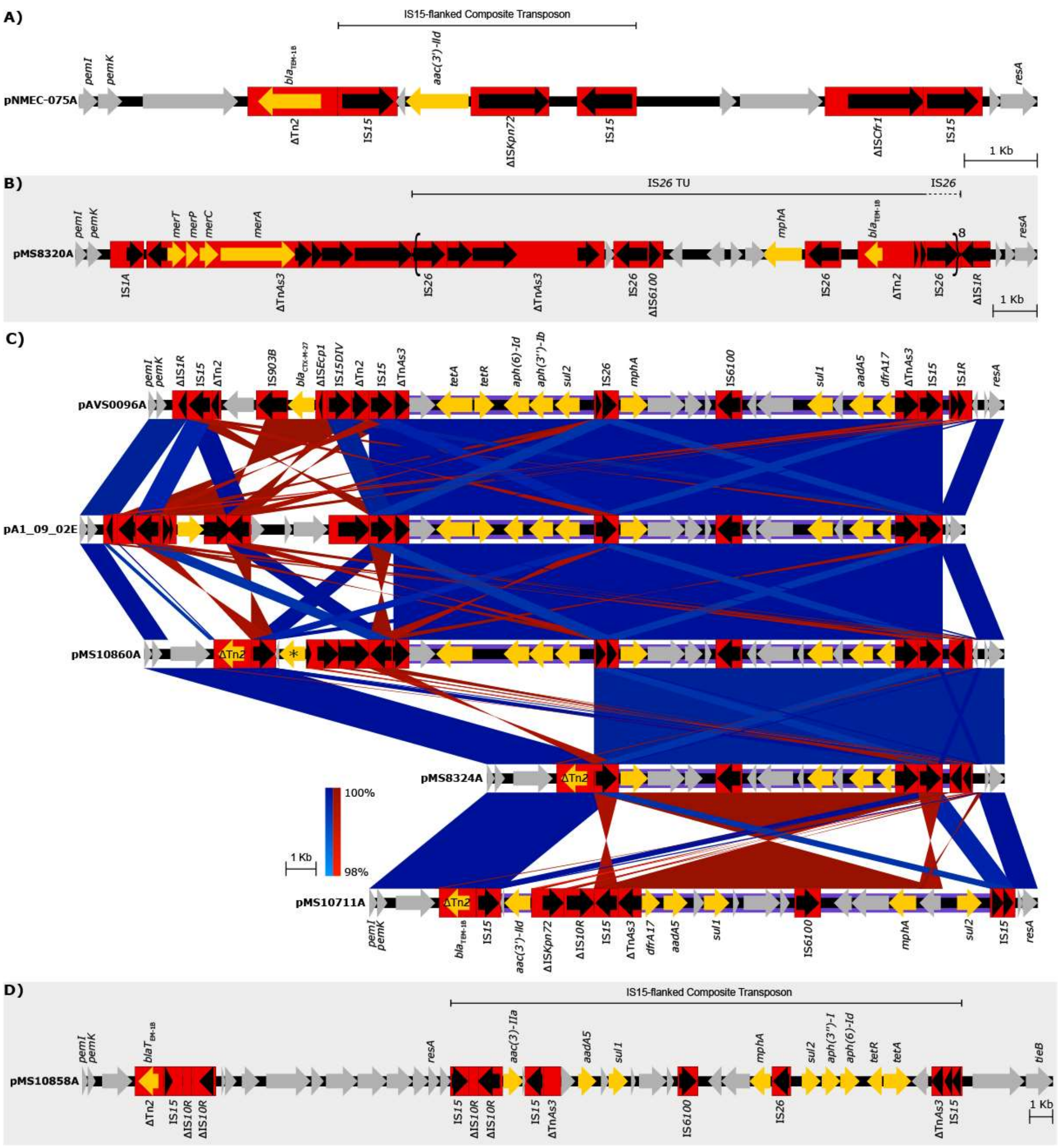

Supplementary Figure S4. Linear schematics of the antimicrobial resistance gene region carried on F-type plasmids in the eight complete *Escherichia coli* ST1193 strains. (A) The small resistance region of pNMEC-075A. The IS*15*-flanked composite transposon is indicated by a solid black line above. (B) Schematic of Tn*7394* on pMS8320A carrying the larger IS*26* transposable unit tandem array variant. Black brackets indicate the tandem array. The IS*26* TU is indicated by a solid black line above, with the dotted black line indicating the shared flanking IS*26*. The smaller IS*26* TU variant integrates at the IS*26* downstream of *mphA*, removing the Tn*2*-element carrying *bla*$_{\text{TEM-1B}}$. (C) Linear nucleotide comparisons of the antimicrobial resistance gene regions of plasmids pAVS0096A, pA1_09_02E, pMS10860A, pMS8324A and pMS10711A. (D) Schematic of the resistance region of pMS10858A. The IS*15*-flanked composite transposon is indicated by a solid black line above. Blue shading indicates nucleotide identity between sequences according to BLASTn. Key genomic regions are indicated: IS: red, IS CDSs: black, antimicrobial resistance genes: yellow, other CDSs: grey. Purple rectangles: resistance islands. Asterisk in pMS10860A indicates *aac(3')-IId*. Blue and red (inversions) shading indicates nucleotide identity between sequences according to BLASTn (98 to 100%). Image created using Easyfig [25].

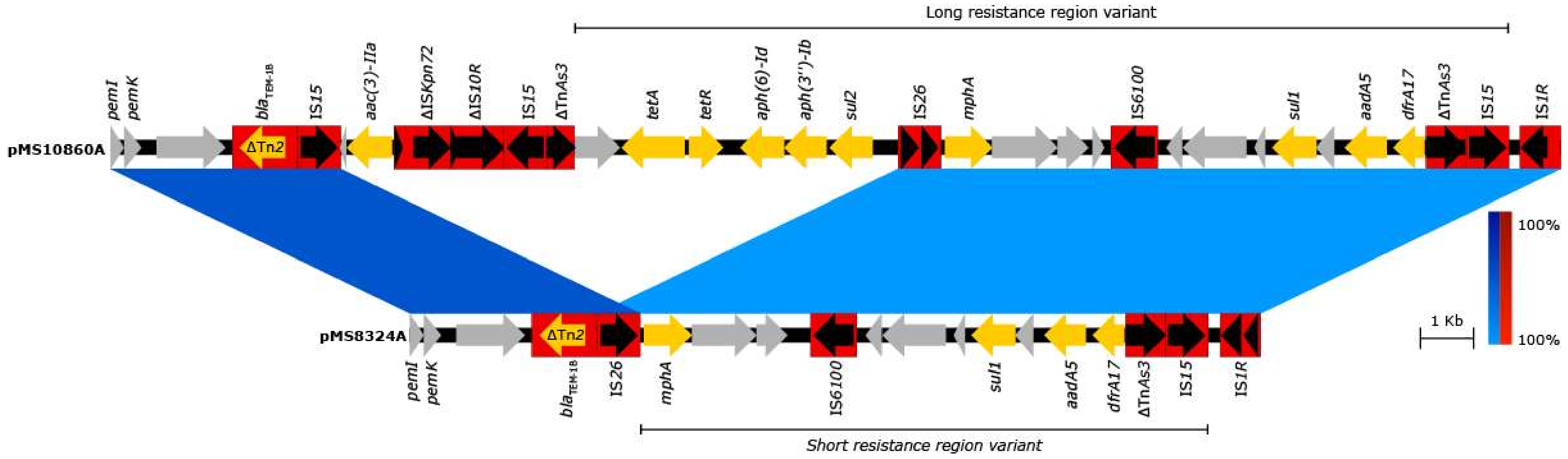

Supplementary Figure S5. Linear nucleotide pairwise comparisons between two *Escherichia coli* sequence type (ST)1193 F-type plasmids highlighting resistance region variants. Plasmids are labelled to the left of the gene sequence. Blue shading indicates nucleotide identity between sequences according to BLASTn. Key genomic regions are indicated: IS: red, IS CDSs: black, antimicrobial resistance genes: yellow, other CDSs: grey. pMS10860A carries the long resistance region variant and pMS8324A carries the short resistance region variant. Blue shading indicates nucleotide identity between sequences according to BLASTn (100%). Image created using Easyfig [25].

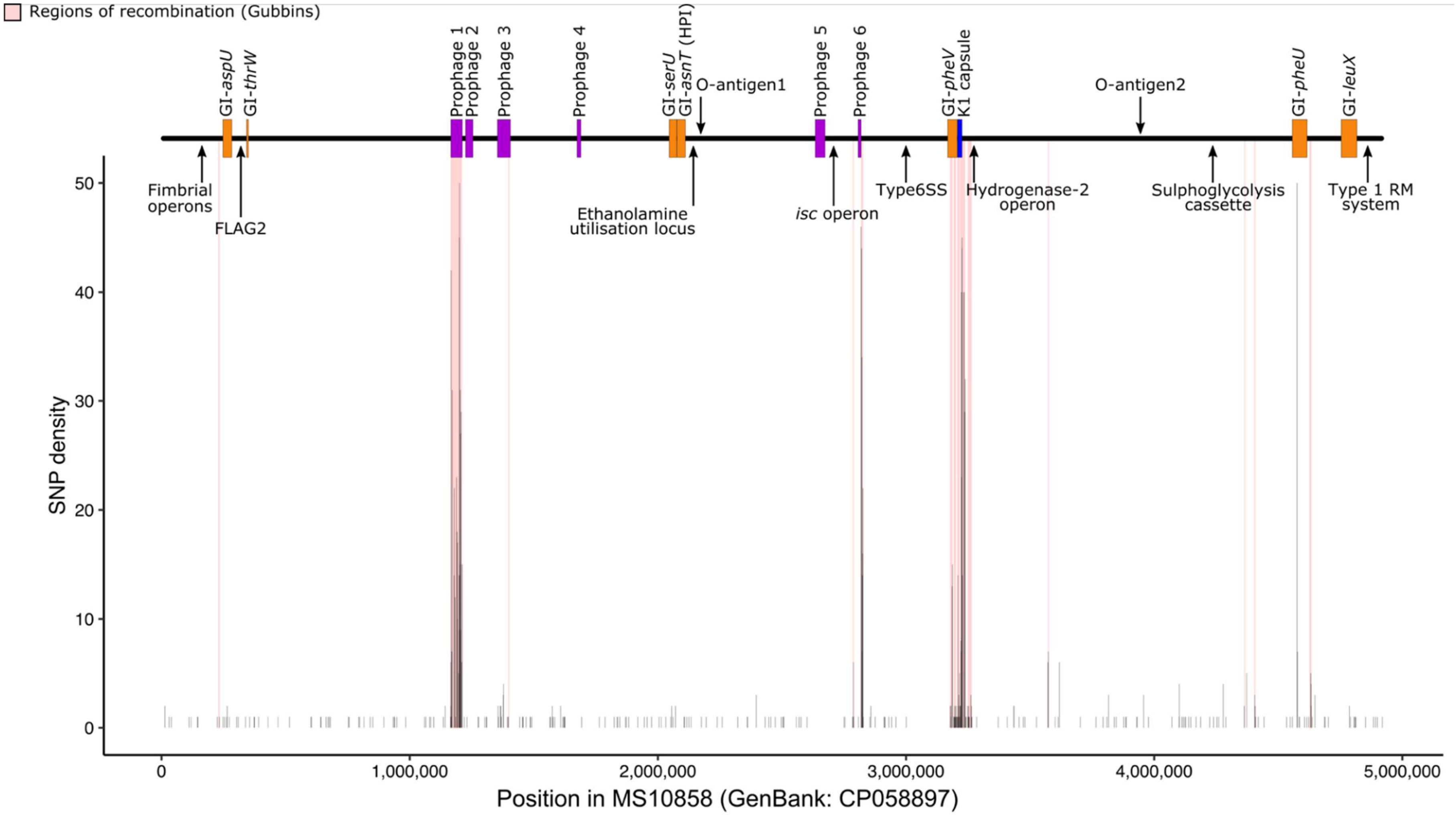

Supplementary Figure S6. Genome-wide single-nucleotide polymorphism distribution against *Escherichia coli* strain MS10858. A total of 3,553 single-nucleotide polymorphisms (SNPs) were derived from a whole-chromosome global alignment (5,399,477 bp) and are called against the reference chromosome MS10858 (GenBank: CP58897). The bar plots represent the number of SNPs in a 1,000 bp window. Genomic islands and prophages are highlighted in orange and purple, respectively.

MS10863 K1(-) K5(+)
MS10860 K1(-) K5(+)
MS10857 K1(-) K5(+)
MS10858 K1(+) K5(-)
MS10854 K1(-) K5(-)
MS10851 K1(+) K5(-)
MS10850 K1(+) K5 (-)
MS10746 K1(+) K5 (-)

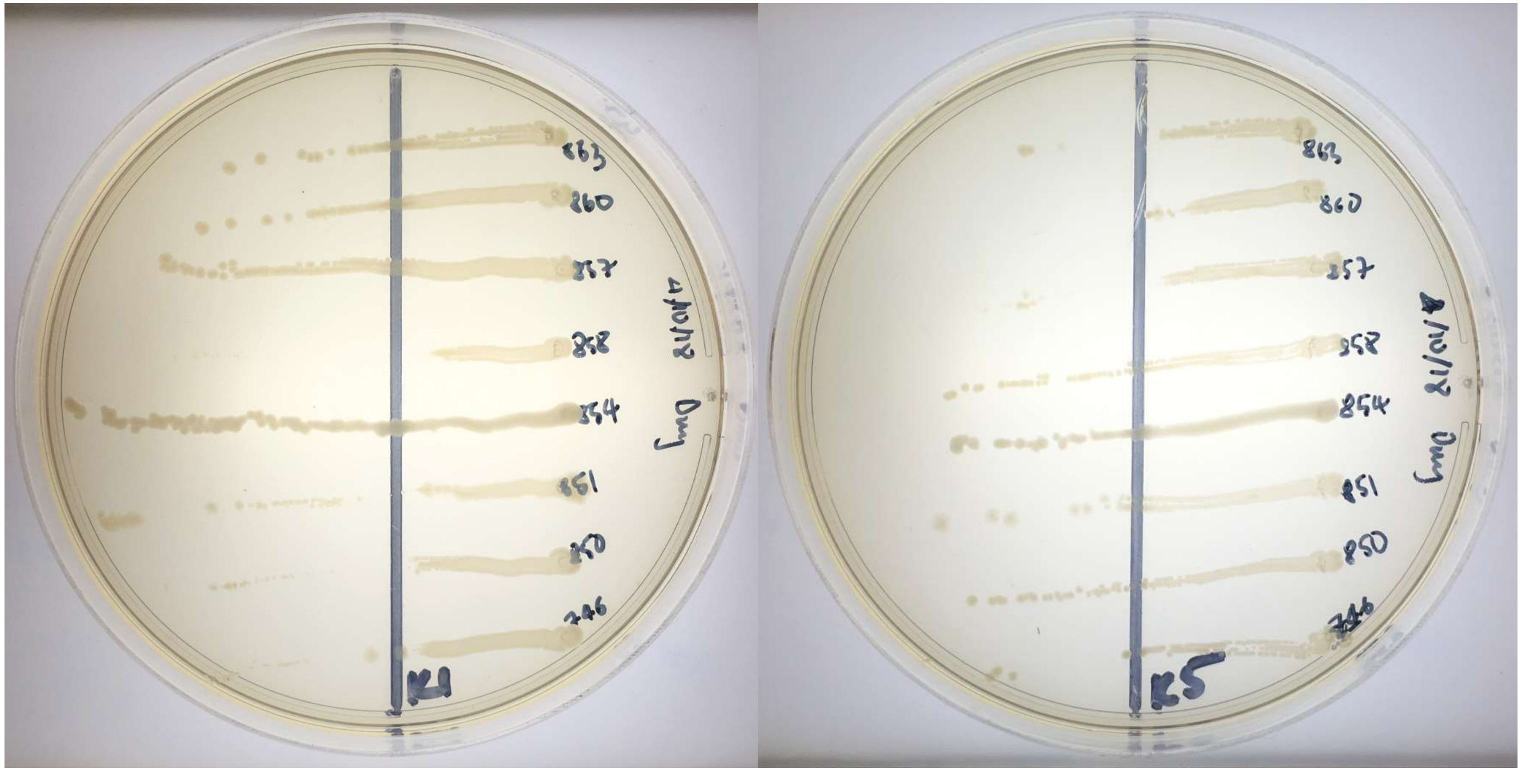

Supplementary Figure S7. Cross-brush agar test results show K1 phage reaction with strain MS10858 and K5 phage reaction with strain MS10860. The vertical line indicates the line of phage suspension.

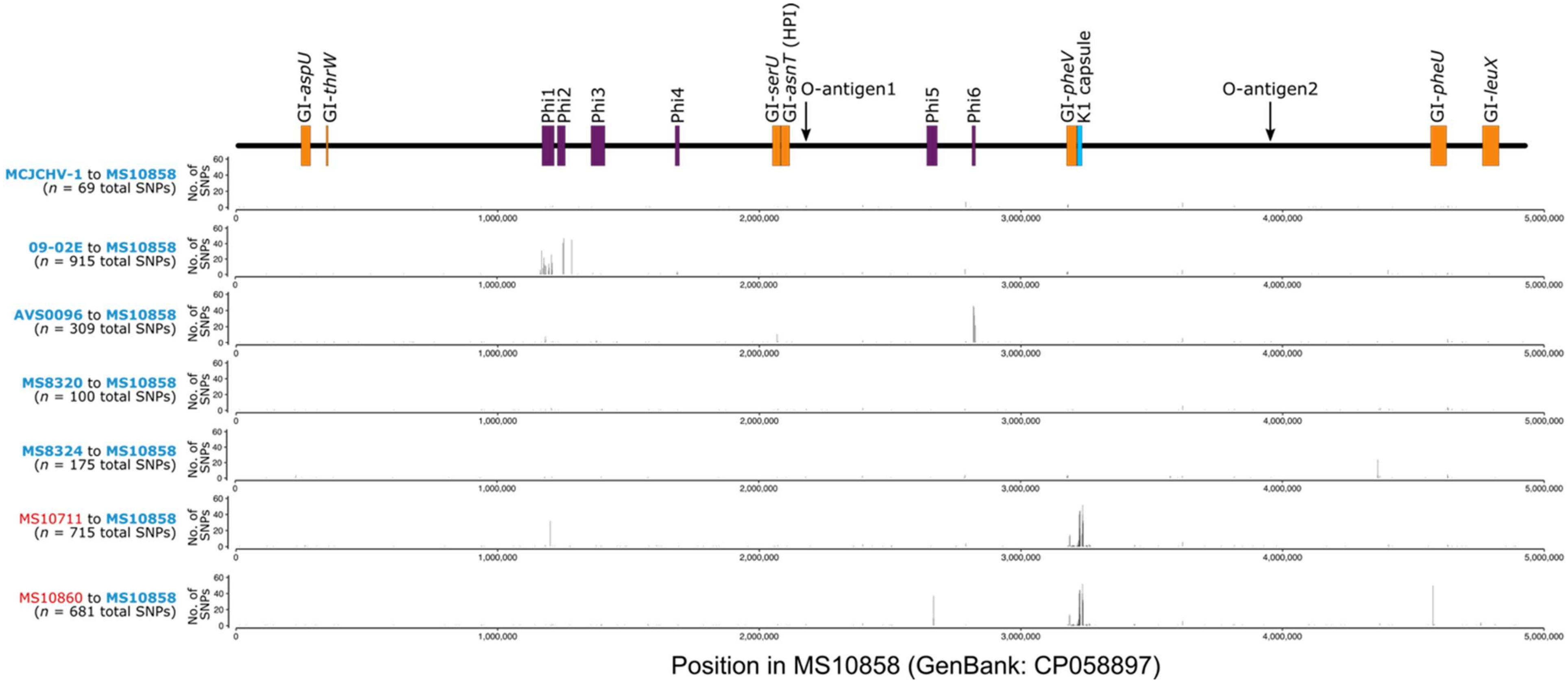

Supplementary Figure S8. Distribution of single-nucleotide polymorphisms (SNPs) across the genome of *Escherichia coli* ST1193 strain MS10858 compared to the genomes of sequence type (ST)1193 strains MS10711, MS10860, MS8320, MS8324, MCJCHV-1, 09-02E, and AVS0096. The histograms represent the number of SNPs in a 1,000 bp window. The horizontal line above the histograms corresponds with key genomic regions in the genome of MS10858. Genomic islands and prophages are highlighted in orange and purple, respectively. Strain names coloured in blue or red represent genomes with a K1 or K5 capsular antigen, respectively. Total SNP count includes prophages, genomic islands and the capsular region and is an overestimate compared with the core genome SNP frequency.

5. References

1. **Wood DE, Salzberg SL**. Kraken: ultrafast metagenomic sequence classification using exact alignments. *Genome Biology* 2014;15:R46 doi: 10.1186/gb-2014-15-3-r46

2. **Sayers EW, Barrett T, Benson DA, Bryant SH, Canese K, Chetvernin V, *et al.*** Database resources of the National Center for Biotechnology Information. *Nucleic Acids Research* 2009;37:D5-D15 doi: 10.1093/nar/gky1069

3. **White RT, Legione AR, Taylor-Brown A, Fernandez CM, Higgins DP, Timms P, *et al*.** Completing the genome sequence of *Chlamydia pecorum* strains MC/MarsBar and DBDeUG: new insights into this enigmatic Koala (*Phascolarctos cinereus*) pathogen. *Pathogens* 2021;10:1543 doi: 10.3390/pathogens10121543

4. **Bolger AM, Lohse M, Usadel B**. Trimmomatic: a flexible trimmer for Illumina sequence data. *Bioinformatics* 2014;30:2114-2120 doi: 10.1093/bioinformatics/btu170

5. **De Coster W, D'Hert S, Schultz DT, Cruts M, Van Broeckhoven C**. NanoPack: visualizing and processing long-read sequencing data. *Bioinformatics* 2018;34:2666-2669 doi: 10.1093/bioinformatics/bty149

6. **Lin Y, Yuan J, Kolmogorov M, Shen MW, Chaisson M, Pevzner PA**. Assembly of long error-prone reads using de Bruijn graphs. *Proceedings of the National Academy of Sciences of the United States of America* 2016;113:E8396-E405 doi: 10.1073/pnas.1604560113

7. **Kolmogorov M, Yuan J, Lin Y, Pevzner PA**. Assembly of long, error-prone reads using repeat graphs. *Nature Biotechnology* 2019;37: 540–546 doi: 10.1038/s41587-019-0072-8

8. **Altschul SF, Gish W, Miller W, Myers EW, Lipman DJ**. Basic local alignment search tool. *Journal of Molecular Biology* 1990;215:403-410 doi: 10.1016/S0022-2836(05)80360-2

9. **Carver TJ, Rutherford KM, Berriman M, Rajandream MA, Barrell BG, Parkhill J**. ACT: the Artemis Comparison Tool. *Bioinformatics* 2005;21:3422-3423 doi: 10.1093/bioinformatics/bti553

10. **Li H, Handsaker B, Wysoker A, Fennell T, Ruan J, Homer N, *et al***. The sequence alignment/map format and SAMtools. *Bioinformatics* 2009;25:2078-2079 doi: 10.1093/bioinformatics/btp352

11. **Walker BJ, Abeel T, Shea T, Priest M, Abouelliel A, Sakthikumar S, *et al***. Pilon: an integrated tool for comprehensive microbial variant detection and genome assembly improvement. *PLOS One* 2014;9:e112963 doi: 10.1371/journal.pone.0112963

12. **Kidsley AK, White RT, Beatson SA, Saputra S, Schembri MA, Gordon D, *et al***. Companion animals are spillover hosts of the multidrug-resistant human extraintestinal *Escherichia coli* pandemic clones ST131 and ST1193. *Frontiers in Microbiology* 2020;11:1968 doi: 10.3389/fmicb.2020.01968

13. **Sarowska J, Futoma-Koloch B, Jama-Kmiecik A, Frej-Madrzak M, Ksiazczyk M, Bugla-Ploskonska G, *et al***. Virulence factors, prevalence and potential transmission of extraintestinal pathogenic *Escherichia coli* isolated from different sources: recent reports. *Gut Pathogens* 2019;11:10 doi: 10.1186/s13099-019-0290-0

14. **Pressler U, Staudenmaier H, Zimmermann L, Braun V**. Genetics of the iron dicitrate transport system of *Escherichia coli*. *Journal of Bacteriology* 1988;170:2716-2724 doi: 10.1128/jb.170.6.2716-2724.1988

15. **Staudenmaier H, Van Hove B, Yaraghi Z, Braun V**. Nucleotide sequences of the *fecBCDE* genes and locations of the proteins suggest a periplasmic-binding-protein-dependent transport mechanism for iron(III) dicitrate in *Escherichia coli*. *Journal of Bacteriology* 1989;171:2626-2633 doi: 10.1128/jb.171.5.2626-2633.1989

16. **Van Hove B, Staudenmaier H, Braun V**. Novel two-component transmembrane transcription control: regulation of iron dicitrate transport in *Escherichia coli* K-12. *Journal of Bacteriology* 1990;172:6749-6758 doi: 10.1128/jb.172.12.6749-6758.1990

17. **Hagan EC, Mobley HL**. Haem acquisition is facilitated by a novel receptor Hma and required by uropathogenic *Escherichia coli* for kidney infection. *Molecular Microbiology* 2009;71:79-91 doi: 10.1111/j.1365-2958.2008.06509.x

18. **Kehres DG, Maguire ME**. Emerging themes in manganese transport, biochemistry and pathogenesis in bacteria. *FEMS Microbiology Reviews* 2003;27:263-290 doi: 10.1016/S0168-6445(03)00052-4

19. **Johnson TJ, Danzeisen JL, Youmans B, Case K, Llop K, Munoz-Aguayo J, *et al***. Separate F-type plasmids have shaped the evolution of the H30 subclone of *Escherichia coli* sequence type 131. *mSphere* 2016;1 doi: 10.1128/mSphere.00121-16

20. **Johnson TJ, Elnekave E, Miller EA, Munoz-Aguayo J, Flores Figueroa C, Johnston B, *et al***. Phylogenomic analysis of extraintestinal pathogenic *Escherichia coli* sequence type 1193, an emerging multidrug-resistant clonal group. *Antimicrobial Agents and Chemotherapy* 2019;63 doi: 10.1128/AAC.01913-18

21. **Roberts RJ, Vincze T, Posfai J, Macelis D**. REBASE--a database for DNA restriction and modification: enzymes, genes and genomes. *Nucleic Acids Research* 2015;43:D298-D299 doi: 10.1093/nar/gku1046

22. **Zankari E, Hasman H, Cosentino S, Vestergaard M, Rasmussen S, Lund O, *et al***. Identification of acquired antimicrobial resistance genes. *Journal of Antimicrobial Chemotherapy* 2012;67:2640-2644 doi: 10.1093/jac/dks261

23. **Stephens C, Arismendi T, Wright M, Hartman A, Gonzalez A, Gill M, *et al***. F plasmids are the major carriers of antibiotic resistance genes in human-associated commensal *Escherichia coli*. *mSphere* 2020;5 doi: 10.1128/mSphere.00709-20

24. **Alikhan NF, Petty NK, Ben Zakour NL, Beatson SA**. BLAST Ring Image Generator (BRIG): simple prokaryote genome comparisons. *BMC Genomics* 2011;12:402 doi: 10.1186/1471-2164-12-402

25. **Sullivan MJ, Petty NK, Beatson SA**. Easyfig: a genome comparison visualizer. *Bioinformatics* 2011;27:1009-1010 doi: 10.1093/bioinformatics/btr039
